# A systematic review of self-regulation measures in children: Exploring characteristics and psychometric properties

**DOI:** 10.1371/journal.pone.0309895

**Published:** 2024-09-19

**Authors:** Yu-Wei Ryan Chen, Nicolas Janicaud, David Littlefair, Pamela Graham, Nicolette Soler, Sarah Wilkes-Gillan, Tomomi McAuliffe, Reinie Cordier

**Affiliations:** 1 Faculty of Medicine and Health, Sydney School of Health Sciences, The University of Sydney, Sydney, NSW, Australia; 2 Nick Janicaud Occupational Therapy, Bondi Junction, NSW, Australia; 3 Faculty of Health & Life Sciences, Department of Social Work, Education and Community Wellbeing, Northumbria University, Newcastle, United Kingdom; 4 School of Health Sciences, College of Health, Medicine and Wellbeing, The University of Newcastle, Newcastle, NSW, Australia; 5 Stepping Stones Therapy for Children, Charlestown, NSW, Australia; 6 School of Health and Rehabilitation Sciences, The University of Queensland, Brisbane, QLD, Australia; 7 Faculty of Health Sciences, Curtin School of Allied Health, Curtin University, Perth, WA, Australia; 8 Faculty of Health Sciences, Department of Health & Rehabilitation Sciences, University of Cape Town, Cape Town, South Africa; University of Maribor, SLOVENIA

## Abstract

Self-regulation, which encompasses cognitive, behavioural, and emotional domains, poses challenges in consistent measurement due to diverse definitions and conceptual complexities. In recognition of its profound impact on long-term mental health and wellbeing in children, this systematic review examined available self-regulation measures for children and young people between 1 and 18 years of age. The systematic review followed the COSMIN taxonomy and reported on the measurement tools’ characteristics and psychometric properties. The methodology and reporting were guided by the Preferred Reporting Items for Systematic Reviews and Meta-Analyses (PRISMA) statement and checklist. The protocol for this review was registered with PROSPERO (Number CRD42020155809). A search of six databases (Embase, MEDLINE, PsycINFO, Scopus, CINAHL and ERIC) was performed, and grey literature was searched to identify studies on the psychometric properties of measures assessing all three domains (cognitive, behavioural, and emotional) of self-regulation. The types of psychometric properties were examined against the COSMIN taxonomy of measurement properties. A total of 15,583 studies were identified, and 48 of these met the criteria that reported psychometric properties of 23 self-regulation measures assessing all three domains of self-regulation. Most measures relied on self-reports for ages 11–17, and all had limited psychometric evaluation. The Emotion Regulation Checklist was the most studied measure. Notably, none of the studies evaluated measurement error. The content validity was inadequately evaluated, particularly in terms of comprehensiveness and comprehensibility. Future research should focus on developing measures for young children, evaluating measurement error, and enhancing content validity for comprehensive understanding and effective intervention.

## Introduction

Self-regulation is a central facet of human functioning [1,2], encompassing a person’s ability to control their behaviour, emotions, thoughts and attention to attain specific goals [3,4]. Previous researchers found that self-regulation is positively associated with school achievement [1,5–7], wellbeing and mental health [1,5]. Without proficient self-regulation skills, building lasting friendships, engaging in healthy romantic relationships and participating in appropriate social roles would be almost unattainable [6,8]. Indeed, children need to develop and apply self-regulation skills successfully across multiple aspects of life [1,9].

However, contrasting perspectives and various disciplinary specialisations have resulted in multiple conceptual meanings for self-regulation [10]. Although conceptually, there are related and overlapping terms for self-regulation (e.g., emotional regulation), inconsistency and incongruency in terminology have led to complexity in defining self-regulation [10,11]. Further, the term self-regulation encompasses varying concepts and consists of multiple facets with no exact indicator of what constitutes self-regulation, further complicating the measurement of its construct [10]. These discrepancies in terminology and definitions of constructs being measured resulted in difficulties in integrating research findings across studies and disciplines.

In addition, timely and effective assessment and identification of self-regulation difficulties are crucial to ensure the implementation of appropriate early interventions to prevent long-term mental health and wellbeing difficulties associated with poor self-regulation [12]. Measuring the outcome of service provision is imperative to discern the value and impact of treatment, regardless of population and practice area [13]. Validated, sensitive and reliable self-regulation measures to assess treatment efficiency in children and adolescents are necessary for clinical use.

To date, very few studies have reviewed self-regulation measures for children. For example, Philpott-Robinson, Johnson [11] conducted a recent scoping review to identify self-regulation assessment tools in 67 studies, highlighting inconsistencies in the measures and constructs used for assessing self-regulation in pre-school and elementary-aged children. Similarly, Solé-Ferrer, Mumbardó Adam [14] systematically reviewed 37 self-regulation measures from 50 studies for children and adolescents, revealing considerable diversity in the measures used, which often attributed to the lack of consensus in the definition of the self-regulation construct. Although a range of self-regulation measures have been identified in these reviews, there was a lack of a well-defined concept of self-regulation for guiding the selection of measurement tools. This, coupled with the omission of reviews of psychometric properties, particularly the critical consideration of content validity [15], hinders the practical and research application of these findings.

There is an urgent need for a systematic review to adopt a universal definition of self-regulation, identify and outline the characteristics of self-regulation measures, and evaluate their psychometric properties. In contemporary literature, self-regulation is a multi-dimensional construct encompassing three specific domains: cognitive, emotional, and behavioural regulation [10,16–18]. Therefore, our study presents a unique perspective compared to previous reviews, focusing on assessing self-regulation across all three domains. Specifically, cognitive regulation involves mental functions such as attention, memory, flexibility and the management of thoughts [16,19,20]. Emotional regulation pertains to managing and influencing emotions, including their expression and experience [21,22]. Behavioural regulation encompasses controlling actions, including the ability to inhibit or initiate behaviours and manage impulses [20]. Thus, our systematic review aimed to: (a) identify the available self-regulation measures used in paediatric populations (0–18 years) from the studies investigating their psychometric properties, (b) summarise the characteristics of the measures and included studies, and (c) evaluate the psychometric properties of these measures, including their development and content validity. The evaluation of psychometric properties follows the terminology and definitions outlined in the COSMIN (**CO**nsensus-based **S**tandards for the selection of health **M**easurement **IN**strument) taxonomy [23].

## Methods

This systematic review was conducted following the Preferred Reporting Items for Systematic Reviews and Meta-Analyses (PRISMA) statement guidelines [24]. The ten items of the *A MeaSurement Tool to Assess systematic Reviews 2 (AMSTAR 2) Field* [25] were also used. The protocol for this review was registered with PROSPERO (Number CRD42020155809).

[The PRISMA checklist is provided as Supporting Information].

### Eligibility criteria

The eligibility criteria for this review encompassed published articles or manuals that provided information on the study of the psychometric properties of measures designed to assess self-regulation. Given the multitude of terms with similar definitions closely related to self-regulation, articles were considered for inclusion if they explicitly claimed to measure any of the terms associated with self-regulation, such as self-regulation, emotion regulation, effortful control, behaviour regulation, or emotional competence. Additionally, articles were included if the measurement tools were applied to populations with an average age below 18 years. However, articles were excluded if the primary focus was not on the measurement of self-regulation but instead utilised the tool to develop another measurement instrument. For instance, an article would be excluded if the self-regulation measure was used solely to establish the criterion validity of another tool not related to self-regulation. In addition, articles were excluded if it was impossible to separate the measurement of self-regulation from other constructs; for example, if a measurement tool claimed to assess both self-regulation and social skills, it would be excluded. Articles written in languages other than English were excluded. Further, conference abstracts, theses, presentations and articles where the measurement tool was unavailable were excluded. We did not restrict the publication date to capture a comprehensive range of measurement tools for our review.

### Information sources

A systematic literature search was performed using medical subject headings (MeSH) or Thesaurus terms and free text on the following online databases: Embase, MEDLINE, PsycINFO, Scopus, CINAHL and ERIC. These databases were selected to cover the wide array of disciplines concerned with self-regulation, such as psychology, education, health sciences and medicine. To identify articles in the databases, we used a combination of different search terms in the following areas: “self-regulation’, ’psychometric properties’, ’assessment’ and ’children’. The original search was performed in February 2023 and updated in March 2024. Grey literature was searched for using Google Scholar. A complete list of search terms and strategies is available in S1 File.

### Study selection

We used Endnote to remove duplicate entries from the systematic literature search across the six databases. We then created the Excel spreadsheet to include the titles and abstracts of the articles retrieved for screening. The second author, who had also provided training to the other reviewer (TM) regarding the eligibility criteria for article inclusion and exclusion, independently reviewed all the titles and abstracts of the retrieved articles against the inclusion criteria. The other reviewer independently evaluated a randomly selected 20% of the article abstracts to ensure rating accuracy. Inter-rater reliability for the screening between the two reviewers was assessed based on weighted Kappa calculations of 0.86 (95% CI = 0.80–0.93). Where disagreements occurred between the reviewers, a third reviewer (YRC) was involved in determining an article’s eligibility for inclusion until a consensus was reached. Due to the high inter-rater reliability achieved [25], the second author screened the remaining articles. In the secondary stage screening, the full-text articles and assessment items were screened against the definitions of the three domains of self-regulation by consensus ratings of the first, second and last authors to ensure the tools satisfied the criteria measuring all three domains (i.e., cognitive regulation [thoughts], emotion regulation [feelings], and behavioural regulation [emotions]). Grey literature was searched using Google Scholar, and reference lists of included studies were searched to identify any articles that were not identified in the systematic literature search.

### Methodological quality of the studies

The methodological quality of each study was systematically assessed using standard quality assessment criteria for evaluating primary research [26]. The Kmet checklist offers a quantifiable measure for evaluating the quality of studies using a 3-point ordinal scale (2 = yes, 1 = partial, 0 = no) applied to 14 criteria, including sampling strategy, justification of analytic methods, and reporting of results. To derive the quality percentage score, the total score is divided by the maximum score, omitting non-applicable criteria. Subsequent classification of methodological quality is then based on the calculated percentage, with scores exceeding 80% classified as strong, those falling within the range of 70–79% classified as good, those between 50–69% classified as fair, and scores below 50% classified as poor.

### Data synthesis

At this stage of analysis, all articles for each of the measurement tools of self-regulation were scrutinised as guided by the Cochrane Handbook for Systematic Reviews [27] and the Systematic Reviews Centre for Reviews and Dissemination [28]. The COSMIN taxonomy [23] was used to identify which psychometric properties have been studied and reported on for each of the measurement tools. We particularly examined the content validity due to its recognised importance as the most psychometric property for investigation in a measurement tool [15]. Comprehensive data forms were developed and populated for the following information:

- Measurement tool characteristics–including the purpose, measurement type, recall period, scale titles and number of items, response options, number of scales and range of scores, and the interpretation of scores.
- Study characteristics–including the purpose of the study, sample size, population characteristics, the age ranges captured in each study and the methodological quality of each study.
- Psychometric properties–reporting on whether a study has been conducted on each of the properties detailed in COSMIN: internal consistency, reliability, measurement error, content validity, structural validity, hypothesis testing, measurement invariance/ cross-cultural validity and criterion validity. The examination of responsiveness was excluded in this study because investigating it requires reviewing studies that have employed the identified measures as an outcome assessment.
- Content validity–reporting on three aspects of the content of an instrument: (a) relevance (i.e., the degree to which all items of a measurement tool are relevant for the construct of interest within a target population and purpose of use), (b) comprehensiveness (i.e., the degree to which all key concepts of the construct are included in a measurement tool), and (c) comprehensibility (i.e., the degree to which items of a measurement tool are easy to understand by respondents) [15].

## Results

### Results of search

**Fig 1:**
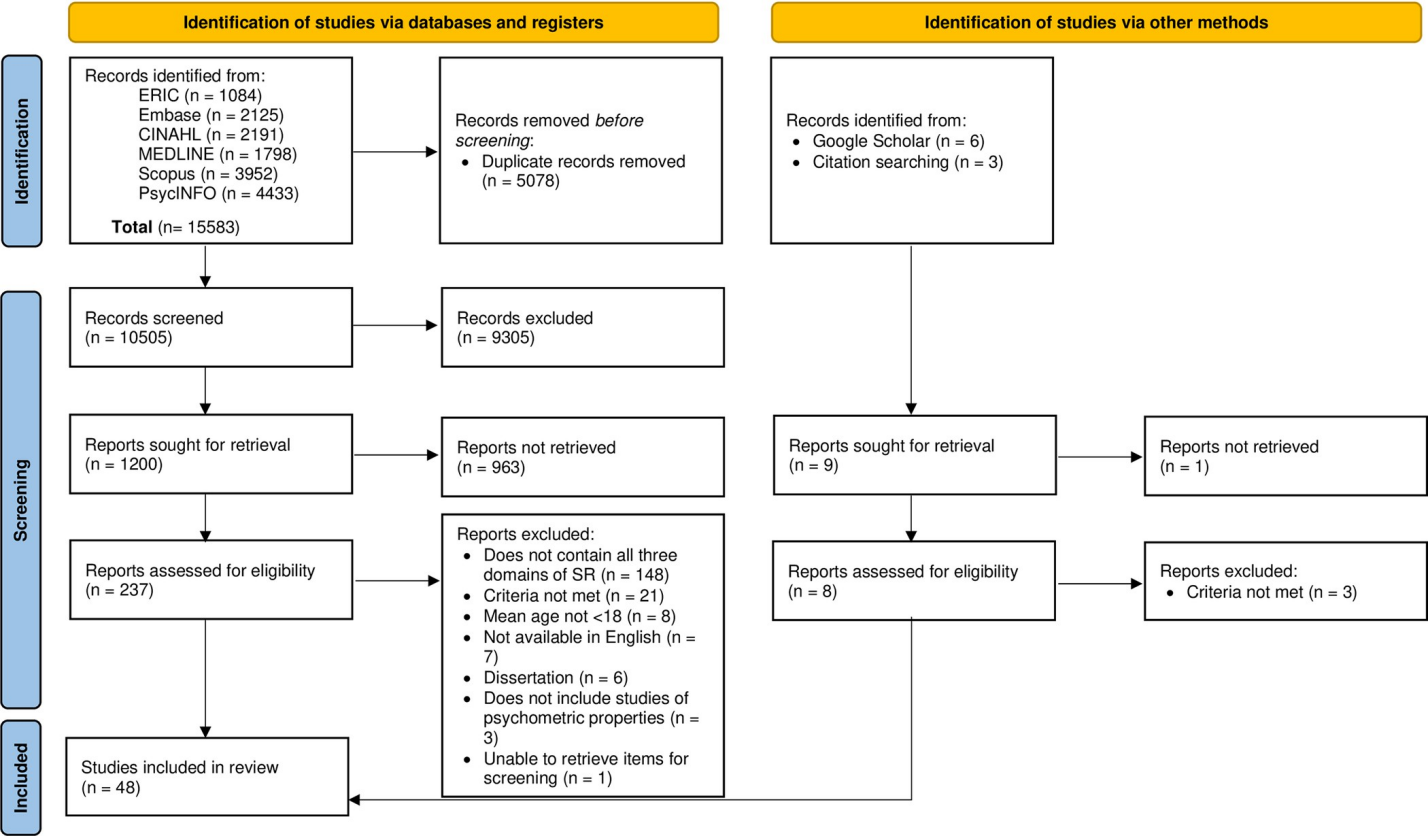
PRISMA flowchart.

The systematic literature search from the six databases produced 15,583 articles. After removing 5,078 duplicates, a total of 10,505 articles were retrieved for screening using the inclusion and exclusion criteria. An additional nine articles were located in total, six via grey literature searching on Google Scholar and three from reference list searching. After screening for titles and abstracts, 237 records were identified for full-text screening, with 48 studies meeting the final eligibility criteria for this review. Fig 1 shows the PRISMA flowchart that illustrates the process of study selection.

### Characteristics of measurement tools

A summary of the characteristics of the identified measurement tools is presented in Table 1. Fourteen of 23 measurement tools were self-report measures, including interviews with the child, where participants were expected to answer questions relating to their self-regulation. The measures used Likert scales, asking participants to rate either how much they agreed that a statement related to them or the frequency of a particular behaviour, thought, or feeling. The scales used were a 5-point (n = 11) or 4-point Likert scale (n = 3). Five measurement tools were parent/carer/teacher reports, where a parent, carer or teacher was asked to answer questions about their child’s self-regulation. Likert scales on the frequency of particular behaviours were used for four measures, while one measure used interviews with parents or carers for assessment. Four measurement tools used observer reports, which were not necessarily from a parent or carer. These measures used Likert scales asking about the degree to which the observer agrees about a child’s behaviour (Pre-school Situational Self-Regulation Toolkit assessment [PRSIST]; Response to Challenge Scale [RCS]) or frequency of behaviours (Pre-school Self-Regulation Assessment [PSRA]; Emotion Regulation Checklist [ERC]). The PSRA also record the latency of a behaviour, levels of waiting on a 4-point Likert scale, and levels of sharing on a 2-point Likert scale. It should be noted that the Difficulties in Emotion Regulation Scale (DERS) was included in the present study in its original form, as well as five other variants where different items were omitted for purposes of validating shortened versions. While all measures except the Adolescent Anger Rating Scale (AARS) were not commercially published, researchers and clinicians must contact the developers for cost information and obtain a copy of the measure.

**Table 1:** Characteristics of measurement tools of self-regulation.

Measurement Tool	Purpose	Measurement Type	Total Items; Subscale (Number of Items)	Response Options	Range of score (R); Interpretation
Adolescent Anger Rating Scale (AARS)	To measure and discriminate reactive and instrumental anger in adolescents	Self-Report	Total # of items: 41 # of Subscales: 3 • Instrumental Anger (20) • Reactive Anger (8) • Anger Control (13)	4-Point Likert 1 = Hardly ever 4 = Very often	R = 41–164 ↑ scores = ↑ frequency of anger behaviour
Adolescent Emotion Regulation Strategies Questionnaire (AERSQ)	A self-report instrument of adolescents’ commonly used ER strategies in daily life	Self-Report	Total # of items: 20 # of Subscales: 5 • Rumination/negative thinking (7) • Positive reorientation (4) • Communication (2) • Distraction (4) • Cultural activities (3)	5-Point Likert 1 = Never 5 = Very often	R = 20–100 ↑ scores = ↑ frequency ER strategies
Adolescent Emotion Regulation Strategies Questionnaire—Extended (AERSQ-E)	An extended version of a self-report instrument of adolescents’ commonly used ER strategies in daily life	Self-Report	Total # of items: 23 # of Subscales: 6 • Rumination/negative thinking (4) • Positive reorientation (4) • Creative expression (4) • Aggressive outlet (4) • Social support (4) • Distraction (3)	5-Point Likert 1 = Never 5 = Very often	R = 23–115 ↑ scores = ↑ frequency ER strategies
Adolescent Self-Regulatory Inventory (ASRI)	A theoretical-based questionnaire that taps regulation in the short- and long-term.	Self-Report	Total # of items: 36 # of Subscales: 2 • Short Term Self-Regulation (13) • Long-Term Self-Regulation (14) Items not included in analysis (9)	5-Point Likert 1 = not at all true for me 5 = really true for me	R = 36–180 ↑ scores = ↑ SR abilities
Children’s Emotion Regulation Processes Survey (CERP)	To assess children’s use of emotion regulation strategies	Parent-Report	Total # of items: 12 # of Subscales: 4 • Problem/emotion-focused (4) • Attention focused (4) • Dominant venting (2) • Submissive venting (2)	7-point Likert 1 = not at all likely 4 = somewhat likely 7 = very likely	R = 12–84 ↑ scores = ↑ frequency of use of the particular emotional regulation strategy
Child Self-Report of Emotional Experience (CSREE)	To evaluate the ability to regulate strong emotions and the children’s perception of parental support around strong emotions	Close-ended interview with the child	Total # of items: 26 # of Subscales: 2 • Close-ended interview-style questions about sadness (13) • Close-ended interview-style questions about anger (13)	4-point Likert 1 = really not like you 2 = sort of not like you 3 = sort of like you 4 = really like you	R = 26–104 ↑ scores = ↑ agreement with a statement about themselves
Difficulties in Emotion Regulation Scale (DERS)	To assess difficulties in emotion regulation among adolescents	Self-Report	Total # of items: 36 # of Subscales: 6 • Non-acceptance (6) • Goals (5) • Impulse (6) • Awareness (6) • Strategies (8) • Clarity (5)	5-point Likert 1 = almost never 0–10% 2 = sometimes 11–35% 3 = about half the time 36–65% 4 = most of the time 66–90% 5 = almost always 91–100%	R = 36–180 ↑ scores = ↑ emotional dysregulation
Difficulties in Emotion Regulation– 8 (DERS-8)	A brief version of the DERS	Self-Report	Total # of items: 8 No subscales	5-point Likert 1 = almost never 2 = sometimes 3 = about half the time 4 = most of the time 5 = almost always	R = 8–40 ↑ scores = ↑ emotional dysregulation
Difficulties in Emotion Regulation Scale– 16 (DERS-16)	A short form of a measure of emotion dysregulation	Self-Report	Total # of items: 16 # of Subscales: 5 1. Non-acceptance (3) 2. Goals (3) 3. Impulse (3) 4. Strategies (5) 5. Clarity (2)	5-point Likert for frequency 1 = almost never 5 = almost always	R = 16–80 ↑ scores = ↑ emotional dysregulation
Difficulties in Emotion Regulation Scale– 18 (DERS-18)	A shorter version that best mirrors the existing, full-length DERS	Self-report	Total # of items: 18 # of Subscales: 6 • Lack of Emotional Awareness (3) • Lack of Emotional Clarity (3) • Difficulties Engaging in Goal-Directed Behaviour When Distressed (3) • Difficulties Controlling Impulsive Behaviours When Distressed (3) • Non-acceptance of Negative Emotional Responses (3) Limited Access to Effective emotional regulation Strategies (3)	5-point Likert for frequency 1 = almost never 2 = sometimes 3 = about half the time 4 = most of the time 5 = almost always	R = 18–90 ↑ scores = ↑ emotional dysregulation
Difficulties in Emotion Regulation–Short Form (DERS-SF)	A short form of the DERS (the DERS-SF)	Self-Report	Total # of items: 18 # of Subscales: 6 • Strategies (3) • Non-acceptance (3) • Impulse (3) • Goals (3) • Awareness (3) • Clarity (3)	5-point Likert 1 = almost never 2 = sometimes 3 = about half the time 4 = most of the time 5 = almost always	R = 18–90 ↑ scores = ↑ emotional dysregulation
Difficulties in Emotion Regulation Scale–Parent Report (DERS-PR)	A parent form-report form of a measure of emotion dysregulation	Parent report	Total # of items: 36 (35 remaining following CFA) Subscale: NR	5-point Likert for frequency 1 = almost never 2 = sometimes 3 = about half the time 4 = most of the time 5 = almost always	R = NR ↑ scores = ↑ emotional dysregulation
Emotional Competence Assessment Questionnaire (ECAQ)	A measure of emotional competence for children	Teacher Report	Total # of items: 30 # of Subscales: 3 • Awareness (9) • Regulation (12) • Wellbeing (9)	6-point Likert for frequency 1 = Never 6 = Always	R = 30–180 ↑ scores = ↑ emotional competence
Early Development of Emotional Competence (EDEC)	To measure the emotional competence of children with complex communication needs	Interview with parent/carer	Total # of questions: 24 Section 1. Temperament/behavioural characteristics (10) Section 2. Questions about children with complex communication needs and family dynamics (14)	NA	NA
Emotion Regulation Checklist (ERC)	To assess processes central to emotionality and regulation in children	Observer-Report	Total # of items: 24 # of Subscales: 2 • Emotion Regulation (8) • Lability/Negativity (15)	4-point Likert 1 = Never 2 = Sometimes 3 = Often 4 = Almost Always	R = 24–96 ↑ scores = ↑ dysregulation
Emotional Skills & Competence Questionnaire (ESCQ)	A self-report measure of emotional intelligence	Self-Report	Total # of items: 45 # of Subscales: 3 • Perceive and Understand emotions (15) • Express and Label emotions (14) • Manage and Regulate emotions (16)	5-Point Likert 1 = Never 5 = Always	R = 45–225 ↑ scores = ↑ emotional competence
Emotional Skills & Competence Questionnaire–Children (ESCQ-C)	A measure of younger children’s emotional intelligence	Self-Report	Total # of items: 21 # of Subscales: 3 • Perceive and Understand emotions (5) • Express and Label emotions (5) • Manage and Regulate emotions (11)	5-Point Likert 1 = Never 5 = Always	R = 21–105 ↑ scores = ↑ emotional competence
Pre-school Self-Regulation Assessment (PSRA)	To assess pre-schoolers’ self-regulatory skills	Direct Assessment & Observer Report	Structured tasks Total # of items: 10 # of Subscales: 3 • Effortful Control (4) • Executive Control (3) • Compliance (3) Assessor Report Total # of items: 28 No subscales	Structured tasks Latency (seconds), percentage or 2/4-Likert scale Assessor Report First 25 items on 4-point Likert 1 = Never 4 = Always 3 items on yes/no scale	Structured tasks R = NA Assessor report: R = NA ↑ scores = ↑ unregulated behaviour Yes/no scale for the presence of extreme negative behaviours
Pre-school Situational Self-Regulation Toolkit assessment (PRSIST)	To assess children’s cognitive, behavioural, and social-emotional self-regulation	Observer Report	Total # of items: 9 No subscales	7-Point Likert The degree to which the child engages in the described behaviour	R = 7–63 ↑ scores = ↑ self-regulation capacities
Preschool Self-Regulation Questionnaire [Questionario di Valutazione dell’Autoregolazione] (QUVA-p)	To assess child dysregulation at pre-school age	Teacher report	Total # of items: 55 # of Subscales: 4 • Cognitive dysregulation (15) • Behavioral dysregulation (14) • Emotional dysregulation (9) • Socio-relational and adaptive difficulties (17)	3-Point Likert for frequency 0 = not at all/ never 2 = often/ always	R = 0–76 ↑ scores = ↑ self-regulation difficulties
Response to Challenge Scale (RCS)	To measure children’s cognitive, affective and motor regulation in response to a physical challenge course	Observer Report	Total # of items: 16 # of Subscales: 3 • Physical (3) • Cognitive (7) • Affective (6)	7-Point Likert Ratings on bipolar adjectives	R = 16–112 ↑ scores = ↑ self-regulation in response to challenge
Regulation of Emotions Questionnaire (REQ)	To assess the frequency with which adolescents use both functional and dysfunctional emotion regulation strategies	Self-report	Total # of items: 19 # of Subscales: 4 • Internal dysfunctional (5) • Internal-functional (5) • External dysfunctional (5) • External-functional (4)	5-Point Likert for frequency 1 = not at all 5 = always	R = 19–95 ↑ scores = ↑ frequency of particular ER strategy
Self-Regulation Scale (SRS)	A self-report tool to measure self-regulation in adolescents	Self-Report	Total # of items: 26 # of Subscales: 3 • Emotional (9) • Cognitive (9) • Behavioural (8)	4-Point Likert 1 = Never true 4 = Always true	R = 26–104 ↑ scores = ↑ self-regulation skills

*Note*. NR = not reported; NA = not applicable; # = number. Data were extracted by the second author between April and May 2023, and following the update in March 2024.

The age distribution for the available assessment tools was skewed toward older age ranges, with a substantial abundance of measurement tools for ages 12 and above (Table 2). Twelve years of age was the most well-represented category, featuring 16 measurement tools. In contrast, ages 0–2 years had only one measurement tool. The intermediate age groups fell between 3 and 11 years, comprising between 5 and 10 measurement tools. Notably, self-report measurement tools were predominantly used for individuals aged 12 and older, while observational measures were more prevalent for the 3–7 age group. Moreover, parent or carer reports were less frequently utilised across all age ranges.

**Table 2:** Types of and ages studied for measurement tools of self-regulation.

Tool	Age (Year)																	
	0	1	2	3	4	5	6	7	8	9	10	11	12	13	14	15	16	17
**AARS**																		
**AERSQ**																		
**AERSQ-E**																		
**ASRI**																		
CSREE*																		
**CERP**																		
**DERS**																		
**DERS-8**																		
**DERS-16**																		
**DERS-18**																		
**DERS-SF**																		
**DERS P**																		
EDEC*																		
**ERC**																		
									
**ECAQ**																		
**ESCQ**																		
**ESCQ-C**																		
**PSRA**																		
**PRSIST**																		
**QUVA-p**																		
**RCS**																		
**REQ**																		
**SRS**																		
**Self-Report**	0	0	0	0	0	1	1	1	1	1	2	5	12	13	13	13	11	11
**Parent Report**	1	1	1	2	3	3	3	3	3	2	3	2	2	1	1	1	1	1
**Observational**	0	0	0	5	5	6	4	3	2	2	2	2	2	0	0	0	0	0
**Total**	**1**	**1**	**1**	**7**	**8**	**10**	**8**	**7**	**6**	**5**	**7**	**9**	**16**	**14**	**14**	**14**	**12**	**12**

* Completed in a format of interview.

*Note*: Data were extracted by the second authors in May 2023, and following the update in March 2024.

### Characteristics of studies

All of the studies were conducted from 2000 onwards, except for one developmental study related to the ERC in 1997 (Table 3). Of the 48 studies, 33 were conducted within the most recent 10-year period (2014–2023), while the remaining 15 distinct studies were carried out before 2014. The sample sizes of child participants varied from 10 to 2,124, with 23 studies involving more than 500 participants. Most participants were from the USA (n = 18), followed by European countries (n = 17). The remaining participants were from Turkey (n = 5), Asian countries (n = 5), South America (n = 2), and Australia (n = 1). All studies except Jamal, Dzulkarnain [29] have good to strong methodological quality ratings. The most common risks of bias identified in studies rated as fair to good include unclear reporting on participant selection methods and insufficient information on participant characteristics.

**Table 3:** Study characteristics of measurement tools of self-regulation.

Measurement Tool	Study	Purpose of Study	Sample Size (total number [N]; Male [M]; Female [F])	Population and Country of Residence	Ages (range [R]; Mean [M]; Standard Deviation [SD])	Methodological Quality
Adolescent Anger Rating Scale (AARS)	Burney and Kromrey [30]	To develop a rating scale that would measure and differentiate between two subtypes of anger; instrumental and reactive	N = 792 M = NR F = NR	Students from a Florida district, USA.	R = 12–19 M = NR SD = NR	77.3%; Good
	Aslan and Sevincler-Togan [31]	To create a version of the AARS for a different culture (Turkish)	N = 569 M = 263 F = 306	elementary to high school students and university students in Turkey	R = 13–23 M = NR SD = NR	81.8%; Strong
Adolescent Emotion Regulation Strategies Questionnaire (AERSQ)	Zhou, Daukantaitė [32]	To examine the usefulness and psychometric properties of the AERSQ	N = 991 M = 49.7% F = 50.3%	The SOL project–students in Grades 7 and 8 in southern Sweden	Timepoint 1 R = NR M = 13.7 SD = NR	90.9%; Strong
Adolescent Emotion Regulation Strategies Questionnaire—Extended (AERSQ-E)	Rådman, Claréus [33]	To evaluate the psychometric properties of an extended version of AERSQ	N = 1104 M = 48.3% F = 49.2% Undisclosed = 2.47%	Adolescents from across six sub-studies using data from different Swedish adolescent community samples (schools)	R = 12–20 Sample 1: M [SD] = 14.17 [0.96] Sample 2: M [SD] = 14.13 [0.88] Sample3: M [SD] = 14.17 [0.85] Sample 4: M [SD] = 14.37 [0.96] Sample 5: M [SD] = 14.45 [0.97] Sample6: M [SD] = 17.10 [0.91]	90.9%; Strong
Adolescent Self-Regulatory Inventory (ASRI)	Moilanen [34]	To examine the factor structure and validity of the ASRI	N = 169 M = 43% F = 57%	Adolescents from a Suburban school in southern Michigan, USA.	R = 11–17 M = 13.79 SD = 1.79	86.4%; Strong
	Dias, del Castillo [35]	To present the results of the adaptation of the ASRI to Portugal	Study 1 N = 823 M = 320 F = 503	Adolescents from public high schools from the north of Portugal.	R = 14–20 M = 16.38 SD = 1.09	86.4%; Strong
			Study 2 N = 435 M = 187 F = 248	Adolescents from public high schools in Portugal.	R = 14–19 M = 16.06 SD = 1.00	
Children’s Emotion Regulation Processes Survey (CERP)	Rodriguez, Solar [36]	To analyse psychometric properties of the CERP in a sample of Chilean pre-schoolers	N = 483 M = 48.9% F = 51.1%	Pre-schoolers recruited through intentional sampling in Chile	R = 4–6 M = 4.9 SD = 0.5	95.5%; Strong
Child Self-Report of Emotional Experience (CSREE)	Bowie [37]	To examine the reliability and validity of an interview-type questionnaire	N = 129 M = 60 F = 66	Children from the ‘Family Health Project’ (Washington State), USA	R = 5.3–12.7 M = 9 SD = NR	81.8%; Strong
Difficulties in Emotion Regulation Scale (DERS)	Weinberg and Klonsky [38]	To examine the psychometric properties of the DERS in adolescents	N = 428 M = 167 F = 261	High school students in New York, USA	R = 13–17 M = NR SD = NR	81.8%; Strong
	Neumann, van Lier [39]	To examine if the DERS has utility in the assessment of ER difficulties among adolescents in the Netherlands	N = 870 M = 429 F = 441	Secondary school students in Amsterdam, Netherlands	R = 11–17 M = 14.34 SD = 1.6	90.9%; Strong
	Sarıtaş-Atalar, Gençöz [40]	To explore the psychometric properties of the DERS in Turkish adolescents	N = 595 M = 295 F = 300	High school students in Turkey	R = 14–17 M = 15.19 SD = 0.49	86.4%; Strong
	Perez, Venta [41]	To confirm six-factor structure of the DERS in a sample of adolescent in-patients, explore the relationship between different aspects of emotion dysregulation and lifetime non-suicidal self-injury, assess the clinical utility of the DERS in detecting lifetime non-suicidal self-injury status	N = 218 M = 90 F = 128	In-patient admissions for a unit serving adolescents with severe treatment-refractory behaviour, psychiatric, and substance disorders, USA	R = 12–17 M = 15.93 SD = 1.41	95.5%; Strong
	Sousa, Linharelhos [42]	To validate the 36‐items DERS in a sample of Portuguese community adolescents	N = 989 M = 46.5% F = 53.5%	Public school students in Portugal	R = 14–18 M = 15.82 SD = 1.27	90.9%; Strong
Difficulties in Emotion Regulation Scale-8 (DERS-8)	Penner, Steinberg [43]	To develop an even briefer unidimensional measure of the DERS, and evaluate the construct validity of the new measure	*Sample 1* N = 619 M = 35.5% F = 64.5% *Sample 2* N = 200 M = NR F = NR	*Sample 1*: adolescents ages 12–17 who had been admitted to a private inpatient psychiatric hospital. *Sample 2*: randomly selected from study 1	*Sample 1* R = 12–17 M = 15.33 SD = 1.43 *Sample 2* R = 18–25 M = NR SD = NR	90.9%; Strong
Difficulties in Emotion Regulation Scale 16 (DERS-16)	Charak, Byllesby [44]	To examine psychometric properties of alternate forms of the DERS among adolescents and adults with severe mental illness	N = 636 M = 35.5% F = 64.5%	Psychiatric in-patients in Southern United States hospital, USA	R = 12–17 M = 15.33 SD = 1.43	86.4%; Strong
	Demirpence Secinti and Sen [45]	To investigate the reliability and validity of a DERS-16 in a sample of Turkish adolescents	N = 256 M = 50% F = 50%	High School students in Turkey	R = 14–18 M = 15.51 SD = 0.85	81.9%; Strong
Brief Version of Difficulties in Emotion Regulation Scale (DERS-18)	Charak, Byllesby [44]	To examine psychometric properties of alternate forms of the DERS among adolescents and adults with severe mental illness	N = 636 M = 35.5% F = 64.5%	Psychiatric in-patients in Southern United States hospital, USA	R = 12–17 M = 15.33 SD = 1.43	86.4%; Strong
	Rosharudin, Muhammad [46]	To examine the psychometric properties of the Malay version of DERS-18	N = 689 M = 44.6% F = 55.4%	School students across all regions of Malaysia	R = 13–14 M = 13.5 SD = 0.5	90.9%; Strong
	Nooripour, Ghanbari [47]	To examine the Persian validation of DERS-18 and its role in predicting aggression in Iranian adolescents	N = 1117 M = 43.9% F = 56.1%	Adolescents (14–18) from five regions in the north, south, centre, east, and west of Iranian public schools	R = 14–18 For exploratory factor analysis: M = 16.23 SD = 0.87 For confirmatory factor analysis: M = 16.35 SD = 0.93	86.4%; Strong
	Victor and Klonsky [48]	To validate the DERS-18	Study 1 N = 429 M = 164 F = 265	High school students North-Eastern Kentucky, USA	R = 13–17 M = NR SD = NR	77.3%; Good
			Study 2 N = 167 M = 38 F = 129	Psychiatric care in-patients North-Eastern United States, USA	R = NR M = 15.61 SD = 1.42	
Difficulties in Emotion Regulation–Short Form (DERS-SF)	Charak, Byllesby [44]	To examine psychometric properties of alternate forms of the DERS among adolescents and adults with severe mental illness	N = 636 M = 35.5% F = 64.5%	Psychiatric in-patients in Southern United States hospital, USA	R = 12–17 M = 15.33 SD– 1.43	86.4%; Strong
	Kaufman, Xia [49]	To develop and validate a short form of the DERS (the DERS-SF)	Sample 1 N = 84 M = 0 F = 84	Self-injuring (n = 29), depressed with no self-injury history (n = 28), typical control (n = 27), USA	R = 13–18 M = 16.04 SD = 1.24	90.9%; Strong
			Sample 2 N = 131 M = 36.8% F = 63.2%	Adolescents recruited from online survey, USA	R = 13–18 M = 15.3 SD = 1.18	
			Sample 3 N = 59 M = 29.6% F = 70.4%	Adolescent suicide attempters (n = 29), typical control (n = 30), USA	R = 12–20 M = NR SD = NR	
Difficulties in Emotion Regulation Scale–Parent Report (DERS-PR)	Bunford, Dawson [50]	To evaluate the psycho- metric properties of the DERS-P among adolescents with and without ADHD	Study 1 N = 978 M = 51.2% F = 48.2%	Residents with parents working at Amazon’s Mechanical Turk, USA	R = 11–17 M = 13.52 SD = 1.93	86.4%; Strong
			Study 2 N = 78 M = 75.6% F = 24.4%	Adolescents with ADHD, USA	R = 10–14 M = 12.2 SD = 0.91	
Emotional Competence Assessment Questionnaire (ECAQ)	Bartroli, Angulo-Brunet [51]	To develop the ECAQ and provide evidence for its psychometric quality	N = 1088 M = 51.9% F = 48.1%	School students in Barcelona, Spain	R = 3–5 M = NR SD = NR	95.5%; Strong
Early Development of Emotional Competence (EDEC)	Na, Wilkinson [52]	To provide initial data supporting the EDEC	American sample N = 10 M = 4 F = 6	American mothers of typically developing children, USA	R = 0–10 M = 5.33 SD = NR	81.8%; Strong
			Korean Sample N = 10 M = 4 F = 6	Korean mothers of typically developing children, USA	R = 0–10 M = 4.25 SD = NR	
Emotion Regulation Checklist (ERC)	Shields and Cicchetti [53]	To develop Q-scale for emotion regulation and autonomy and examine its psychometric properties	N = 223 M = 142 F = 81	Maltreated (142) and non-maltreated children (80), USA	R = 6–12 M = 9.92 SD = NR	86.4%; Strong
	Molina, Sala [54]	To explore the factor structure and the reliability of the Italian version of the ERC	Study 1: N = 1417 M = 47.7% F = 52.3%	Kindergarten and elementary school students scored by mothers, Italy	R = 3–11 M = 8.08 SD = 2.03	77.3%; Good
			Study 2: N = 910 M = 46.8% F = 53.2%	Kindergarten and elementary school students scored by teachers, Italy	R = 3–11 M = 5.77 SD = 2.26	
	Reis, De Oliveira [55]	To perform the translation and cross-cultural adaptation of the ERC and investigate evidence of the validity of its Brazilian version	N = 561 M = 53.3% F = 46.7%	Children, scored by parents (51.7%) and teachers (48.3%), Brazil	R = 3–12 M = 6.7 SD = 2.7	90.9%; Strong
	Meybodi, Mohammadkhani [56]	To investigate the psychometric properties of the ERC for use in Iran	N = 352 M = 185 F = 168	Pre-school children in diverse socio-economic areas of Tehran	R = 3–6 M = 4.5 SD = 1.1	72.7%; Good
	Danisman, Dereli [57]	To examine the psychometric properties of the ERC for Turkish pre-school children	N = 600 M = 312 F = 288	Public pre-school children in Turkey	R = 4–5 M = NR SD = NR	86.4%; Strong
	Jamal, Dzulkarnain [29]	To translate and validate ERC to Malaysian	N = 10 parents, 50 children M = NR F = NR	NR, Malaysia	R = 6–17 M = NR SD = NR	63.6%; Fair
	Lucas‐Molina, Giménez‐Dasí [58]	To investigate psychometric properties of ERC in Spanish children	Study 1 N = 284 M = 51.7% F = 48.3	Typically developing pre-school children in Spain	R = 2.8–5.9 M = 4.38 SD = 0.9	95.5%; Strong
			Study 2 N = 323 M = 50.8% F = 49.2%		R = 5.8–11.8 M = 8.82 SD = 1.76	
	Silverman, Bennett [59]	To examine the factor structure of the ERC among children with ADHD with/without ODD	N = 328 M = 259 F = 69	Children under 7 with ADHD participating in a study through Florida International University, USA	R = 4–9 M = 6.08 SD = 1.63	95.5%; Strong
	Chennaz, Valente [60]	Examine the psychometric properties of the ERC	N = 245 M = NR F = NR	French and Swiss children who were sighted, visually impaired and blind, France and Switzerland	R = 3–12 M = NR SD = NR	86.4%; Strong
Emotional Skills & Competence Questionnaire (ESCQ)	Costa, Faria [61]	To assess the measurement invariance of the ESCQ in a Portuguese and Croatian context	Portuguese Sample N = 627 M = 47.4% F = 52.6%	Secondary school students in Portuguese	R = 14–21 M = 15.5 SD = 0.76	90.9%; Strong
			Croatian Sample N = 562 M = 32.8% F = 67.2%	Secondary school students in Croatia	R = 14–19 M = 16.3 SD = 1.07	
	Faria, Lima Santos [62]	Cross-cultural validation of the ESCQ	Croatian study N = 2124 M = NR F = NR	Students from a High School, University and supervisors from an electronics factory, Croatia	R = NR M = NR SD = NR	86.4%; Strong
			Portuguese study N = 730 M = 39% F = 61%	Secondary school and university students, Croatia	R = 15–18+ M = NR SD = NR	
	Schoeps, Tamarit [63]	To provide further evidence of the factorial structure of ESCQ	N = 1300 M = 46.5% F = 53.5%	Students of schools in the Valencian Community, Spain	R = 12–15 M = 13.47 SD = 1.09	90.9%; Strong
	Faria and Lima-Santos [64]	Validation of the ESCQ in the Portuguese academic context	N = 730 M = 39% F = 61%	High school (n = 381) and university students (n = 349), Portugal	R = 15–18+ M = NR SD = NR	90.9%; Strong
Emotional Skills & Competence Questionnaire–Children (ESCQ-C)	Mohorić [65]	To develop and validate children’s form of the ESCQ	N = 639 M 304 F = 335	Elementary school children in Croatia	R = 10–13 M = 11.24 SD = 0.71	86.4%; Strong
Pre-school Self-Regulation Assessment (PSRA)	Smith-Donald, Raver [66]	To provide preliminary evidence of validity of PSRA battery (including PSRA-AR)	N = 63 M = 29 F = 34	Children attending Head Starts in Chicago, USA.	R = 3–5 M = 5.04 SD = 0.59	86.4%; Strong
					

	Tanribuyurdu and Yildiz [67]	To conduct the validity and reliability studies of the PSRA in Turkey	N = 233 M = 119 F = 114	Pre-schoolers in Ankara, Turkey.	R = 4–5 M = NR SD = NR	90.9%; Strong
	Pangestuti, Kadiyono [68]	To examine the validity and reliability of the PSRA in Indonesia	N = 179 M = NR F = NR	Pre-school children in Banten and West Java, Indonesia	R = 6–7 M = NR SD = NR	77.3%; Good
	Daneri, Sulik [69]	To explore the validity of PSRA-AR for use in socioeconomically diverse and racially/ethnically diverse populations	N = 697 M = 48% F = 52%	Children attending public pre-K in a large, urban setting, USA	R = 3–4 M = 4.42 SD = NR	95.5%; Strong
	Bassett, Denham [70]	To examine the properties of the PRSA	N = 313 M = NR F = NR	Pre-schoolers in North Virginia, USA.	R = 3 M = 3.46 SD = 0.33	90.9%; Strong
					R = 4 M = 4.47 SD = 0.3	
Pre-school Situational Self-Regulation Toolkit assessment (PRSIST)	Howard, Neilsen-Hewett [71]	To explore the viability of the PRSIST	N = 80 M = 41 F = 39	Pre-school children in Australia	R = 3–5 M = 4.46 SD = 0.73	77.3%; Good
Preschool Self-Regulation Questionnaire [Questionario di Valutazione dell’Autoregolazione] (QUVA-p)	Scionti, Luzi [72]	To examine psychometric properties of the QUVA-p	N = 413 M = 48.7% F = 51.3%	Kindergarten children in the cities of Milan and Novara (Northern Italy). Scored by 27 female kindergarten teachers	R = 3–6 M = 4.66 SD = 0.86	81.8%; Strong
Response to Challenge Scale (RCS)	Lakes [73]	Report on psychometric properties of scores obtained using RCS	N = 207 M = NR F = NR	Kindergarten to Grade 5, USA	R = Kindergarten to Grade 5 M = NR SD = NR	86.4%; Strong
	Lakes [74]	Present the development and examine the construct validity of the RCS	N = 198 M = 96 F = 102	Kindergarten to Grade 5 students at private elementary school in Midwest, USA.	R = Kindergarten to Grade 5	81.8%; Strong
Regulation of Emotions Questionnaire (REQ)	Phillips and Power [75]	To report on the development and use of REQ	N = 225 M = 105 F = 119 Other = 1	A wide group of socio-economic groups in the UK	R = 12–19 M = 15.06 SD = NR	77.3%; Good
Self-Regulation Scale (SRS)	Gajda, Małkowska-Szkutnik [76]	To adapt and validate the SRS in a Polish adolescent sample	N = 392 M = 43.1% F = 56.9%	Adolescents in Poland	R = 11–17 M = 14.33 SD = 1.77	81.8%; Strong

*Note*: The information from each study was extracted by the first and second authors between March and April 2023, and following the update in March 2024.

### Psychometric properties of measurement tools

The ERC is the most extensively studied tool, with nine studies conducted between 1997 and 2022, followed by the DERS and PRSA, which have been the subject of five studies, respectively (Table 3). Table 4 provides an overview of the psychometric properties examined in the included measurement tools. Regarding reliability, internal consistency has been assessed for 18 out of 23 measurement tools. However, both inter-rater and test-retest reliability were only examined for ERC and PSRA. Measurement error was not investigated for any of the tools.

**Table 4:** Psychometrics available for measurement tools of self-regulation (except content validity).

Measurement Tool	Reliability		Validity			
	Internal Consistency	Reliability	Structural Validity	Hypothesis Testing	Measurement Invariance	Criterion Validity
AARS	• *α* = .70-.83 [30]	• Test-retest: *r* = .58-.65 [30] • Test-retest in Turkish form: no sig difference between the two administrations [31]	• Three factors by CFA: RMSEA = 0.08 [30] • All items retained in the Turkish form [31]	• Implicit hypotheses tested: discriminant validity and identification of group differences in gender, race, grade level and types of education [30]	• No significant differences between scores of English and Turkish forms [31]	NR
AERSQ	• *α* = .54-.83 [32]	NR	• Five factors by CFA: CFI = 0.895, RMSEA = 0.053, SRMR = 0.053 [32]	• Implicit hypotheses tested: gender difference, correlation with other studied constructs/ measures [32]	NR	NR
AERSQ-E	• *α* = .57-.71 [33]	• Test-retest: *r* = .71- .77 [33]	• Six factors by CFA: CFI = 0.920, RMSEA = 0.053, SRMR = 0.061 [33]	• >75% explicit hypotheses confirmed: correlation with other studied constructs/measures [33]	• Metric and scalar invariance examined [33]	NR
ASRI	• *α* = .73-.91 [34] • *α* = .72-.84 in the Portuguese version [35]	NR	• Two factors by CFA: CFI = 0.88, RMSEA: 0.05, SRMR = 0.07 [34] • Two Factors by CFA in Portuguese version: CFI = 0.69, RMSEA = 0.054 [35]	• >75% explicit hypotheses confirmed: concurrent (i.e., correlation with comparison measure and with constructs theoretically tied to self-regulation) and incremental validity [34] • Implicit hypotheses tested in Portuguese version: correlation with other studied constructs/measures [35]	• No correlation with age but with gender in the Portuguese version [35]	NR
CERP	• *α* >.90 in the Chilean version [36]	NR	• Four factors by CFA in the Chilean version: KMO = 0.89 [36]	• Implicit hypotheses tested in the Chilean version: correlation between different reporters and with other studied constructs/measures [36]	NR	NR
CSREE	• *α* = .69-.89 [37]	NR	• Two constructs by PCA [37]	• >75% explicit hypotheses confirmed: correlation with other studied constructs/measures, predictive validity [37]	NR	NR
DERS	• *α* = .76-.93 in the original version [38] • *α* = .72-.87 in Dutch version [39] • *α* = .60-.91 in Turkish version [40] • *α* = .72-.93 in Portuguese version [42]	NR	• Six factors by EFA in the original version [38] • Six factors by CFA in Dutch version: CFI = 0.92, RMSEA = 0.045 [39] • Six factors by CFA in Turkish version: CFI = 0.91, RMSEA = 0.05, SRMR = 0.07 [40] • Six factors by CFA in inpatients: CFI = 0.95, RMSEA = 0.08 [41] • Six factors by CFA in Portuguese version: CFI = 0.953, RMSEA < 0.06 [42]	• Implicit hypotheses tested: gender difference, correlation between different reporters and with other studied constructs/variables in the original version [38] • >75% explicit hypotheses confirmed in the Dutch version and with in-patients: gender difference and correlation with other studied constructs/variables [39,41] • Implicit hypotheses tested: gender difference and correlation with other studied constructs/variables in Turkish and Portuguese versions [40,42]	• Metric invariance in gender in Dutch and Turkish versions [39,40] • Configural invariance demonstrated in the Portuguese version [42]	NR
DERS-8	NR	NR	• Unidimensional model by Item Response theory (IRT): RMSEA = 0.06 [43]	• >75% explicit hypotheses confirmed, same as the other DERS versions: correlation with other studied constructs/variables [43]	NR	• Same pattern of correlations with studied variables and estimates of reliability as DERS [43]
DERS-16	• *α* = .69-.92 in Turkish version [42]	NR	• Five factors by CFA: CFI = 0.97, RMSEA = 0.10 [44] • Five factors by CFA in Turkish version: CFI = 0.934, RMSEA = 0.078, SRMR = 0.067 [45]	NR	• Metric invariance [44]	• Sig correlations with DERS subscales [45]
DERS-18	• *α* = .62-.82 in Malay version [46] • *α* =. 82 in Persian version [47] • *α* = .77-.91 in the original version [48]	• Test-retest in Persian version: *r* = .83 [47]	• Six factors by CFA: CFI = 0.99, RMSEA = 0.04 [44] • Five factors (Removing Awareness) by CFA in Malay version: CFI = 0.977, RMSEA = 0.049 [46] • Six factors by CFA in Persian version: CFI = 0.94, RMSEA = 0.065, SRMR = 0.05 [47] • Six factors by EFA in the original version [48]	• Implicit hypotheses tested in Malay, Persian and original versions: correlation with other studied constructs/variables and predictive validity [46,47, 48]	• Metric invariance [44]	• Sig correlations with DERS subscales [48]
DERS-SF	• *α* = .79-.91 [49]	NR	• Six factors by CFA: CFI = 0.99, RMSEA = 0.05 [44] • Six factors by CFA: CFI = 0.96, RMSEA = 0.06, SRMR = 0.05 [49]	• Implicit hypotheses tested: correlation with other studied constructs/variables [49]	• Metric and scalar invariance [44]	Sig correlations with DERS subscales [49]
DERS-PR	• *α* = .88-.91 in national sample [50] • *α* = .84-.91 in ADHD sample [50]	NR	• Four factors by CFA: CFI = 0.93, RMSEA = 0.084 [50]	• Implicit hypotheses tested: age and gender difference, correlation with other studied constructs/variables and incremental validity [50]	NR	Sig correlations with DERS subscales [50]
ECAQ	• *α* = .90 [51]	• Test-retest: *r* = 0.78 [51]	• Three specific factors by CFA: CFI = 0.99, RMSEA = 0.097 [51]	• >75% explicit hypotheses confirmed: correlation with other studies construct/variables [51]	NR	NR
ERC	• *α* = .76-.93 in the initial version [53] • *α* = .59-.79 in the Italian version [54] • *α* = .86 in Brazilian version [55] • *α* = .57-.81 in Persian version [56] • *α* = .88-.98 in Turkish version [57] • *α* = .69-.78 in Malaysian version [29] • *α* = .77-.88 in Spanish version [58] • *α* = .62-.83 in ADHD [59] • *α* = .65-.83 in visual impaired [60]	• Inter-rater in initial version: *r* = .56 [53] • Inter-rater in Brazilian version: *r* = .18-.24 [55] • Test-retest in Persian version: *r* = .68-.84 [56] • Test-retest in Turkish version: *r* = .83-.91 [57] • Test-retest in Malaysian version: *r* = .76- .81 [29]	• Two factors by CFA in Italian version: CFI = .93, RMSEA = .064 [54]• Two factors by EFA in Brazilian version: Bartlett X^2^ = 3734.2, *df* = 253, *p* < .001, KMO = .880 [55] • Two factors by EFA in Persian version: Bartlett X^2^ = 1860, *df* = 276, *p* < .001 [56]• Two factors by CFA in Turkish version: CFI = .97, RMSEA = .07, NFI = .95, GFI = .93, AGFI = .90, IFI = .97, SRMR = .06 [57]• Two factors by CFA in Spanish version: CFI = .921, RMSEA = .090 (pre-school children) CFI = .960, RMSEA = .071 (elementary school children) [58]• Two factors by CFA in ADHD: CFI = .575, RMSEA = .062, SRMSR = .108, TLI = .531 [59]	• Implicit hypothesis tested in initial version: correlation with other metrics/constructs [53] • Implicit hypothesis tested in Brazilian, Persian and Turkish: correlation with other metric/construct [55–57] • >75% explicit hypotheses confirmed: correlation with other metrics/constructs in Spanish version [58] • Implicit hypothesis tested: diagnostic difference [59] • >75% explicit hypotheses confirmed: diagnostic and age difference [60]	• No correlation between age and gender in the Persian version [56] • Configural invariance in the Spanish version [58]	NR
ESCQ	• *α* = .83-.88 in Portuguese and Croatian versions [61] • *α* = .64-.92 in Croatian and Portuguese versions [62] • *α* = .79-.90 in the Spanish version [63] • *α* = .67-.89 in the Portuguese version [64]	NR	• Three factors by CFA in Portuguese and Croatian versions: CFI = .939, RMSEA = .047 (Portuguese) and CFI = .957, RMSEA = .04 (Croatian) [61] • Three factors by CFA in the Croatian version: GFI = .87, AGFI = .86, RMSEA = .055; three factors identified by PCA in the Portuguese version [62] • Three factors by CFA in Spanish version: CFI = .93, RMSEA = .055 [63] • Two factors by CFA in Portuguese version: CFI = .92, RMSEA = .09 [64]	• >75% explicit hypotheses confirmed: correlation with other metrics/constructs in Spanish version [63]	• Configural, Metric and Scalar invariance [61] • Partial scaler invariance [63]	NR
ESCQ-C	*α* = .61-.82 [65]	NR	• Four factors by CFA: CFI = .916, RMSEA = .050 [65]	• Implicit hypothesis tested: correlation with other metrics/constructs [65]	NR	NR
PSRA	• *α* = .54-.58 [66] • *α* = .80- .88 Turkish version [67] • *α* = .95 Indonesian version [68]	• Inter-rater: ICC > .8 [66]• Test-retest in Turkish version: *r*_s_ = .86 [67] • Test-retest: *r* = 0.61–0.69 [70]	• Two factors by PCA [66] • Two factors by CFA in the Turkish version: CFI = .90, RMSEA = .11 [67] • Five factors by CFA in the Indonesian version: CFI = .094, RMSEA = .030 [68] • Three factors by CFA in PSRA-AR: CFI = .97, RMSEA = .043 [69] • Two factors by CFA: Across 2 time points CFI = 1.0, RMSEA = 0.0 [70]	• >75% explicit hypotheses confirmed: correlation with other metrics/constructs [66] • Implicit hypothesis tested: correlation with other metrics/constructs [69] • >75% explicit hypotheses confirmed: gender, age, socio-economic risk differences and metrics/constructs [70]	• No differences between boys and girls [66] • Configural, metric and scalar invariance [69]	NR
PRSIST	NR	NR	• Two factors by PCA [71]	• >75% explicit hypotheses confirmed: correlation with age and other metrics/constructs [71]	NR	NR
QUVA-p	• *α* = .80-.93 [72]	NR	• Three factors by CFA: CFI = .916, RMSEA = .051 [72]	• Implicit hypothesis tested: correlation with other metrics/constructs [72]	• Configural, metric and scalar invariance [72]	NR
RCS	NR	NR	• Two-facet generalizability analysis identified the differentiation of three domains [74]	• >75% explicit hypotheses confirmed: correlation with other metrics/constructs [73]	NR	NR
REQ	• *α* = .66-.76 [75]	NR	• Four factors by PCA and SEM [75]	• >75% explicit hypotheses confirmed: correlation with other metrics/constructs [75]	NR	NR
SRS	• *α* = .83-.86 [76]	NR	• Three factors by CFA: CFI = .93, RMSEA = .051 [76]	• Implicit hypothesis tested: correlation with other metrics/constructs [76]	NR	NR

*Note*. NR = not reported; EFA: exploratory factor analysis; CFA: confirmatory factor analysis; PCA: principal components analysis; SEM: structural equation modelling; Cross-cultural validation has been integrated with other domains of psychometric properties; Measurement error was not examined across the included studies; EDEC was not included in the table as only cross-cultural translation and content validity was reported. Data were extracted by the First author in July 2024.

In terms of validity, the structural validity of all measurement tools except for Early Development of Emotional Competence (EDEC) has been evaluated primarily through factor analysis. Hypothesis testing was conducted for all tools except Difficulties in Emotion Regulation Scale 16 (DERS-16) and EDEC. Eleven measurement tools have been examined for cross-cultural validity in different languages. ERC is the most widely used in cross-cultural studies, spanning six different countries in addition to the USA. Furthermore, measurement invariance, which is the other aspect of cross-cultural validity (e.g., differences across age groups or client populations), was explored in ten measures. The criterion validity of other versions of DERS was examined against the original DERS.

Concerning content validity, most measurement tools have been examined for their relevance, with each tool being developed based on a stated definition of the self-regulation construct (Table 5). However, it remains unclear whether the development of the construct in the PRSIS was based on a specific theory, conceptual framework, or model. Additionally, the target population for which the development was intended was not clearly disclosed in four measurement tools (Child Self-Report of Emotional Experience [CSREE], DERS-8, DERS-18, Emotional Skills & Competence Questionnaire [ESCQ]). Only six tools included cognitive interview studies or pilot testing as part of their development process. Furthermore, only three studies reported on comprehensibility (DERS-16, Emotional Competence Assessment Questionnaire [ECAQ], PSRA) and three on comprehensiveness (Adolescent Emotion Regulation Strategies Questionnaire [AERSC], ECAQ, PSRA) of the measurement tools from the client’s perspectives.

**Table 5:** Content validity of measurement tools of self-regulation.

	AARS	AERSQ	AEPSQ-E	ASRI	CERP	CSREE	DERS	DERS-8	DERS-16	DERS-18	DERS-SF	DERS-PR	ECAQ	EDEC	ERC	ESCQ	ESCQ-C	PSRA	PRSIST	QUVA-p	RCS	REQ	SRS
**A. Item generation (Relevance)**																							
Is a definition of the construct stated?	X	X	X	X	X	X	X	X	X	X	X	X	X	X	X	X	X	X	X	X	X	X	X
Is a specific theory, conceptual framework or model stated in the development of the construct to be measured?	X	X	X	X	X	X	X	X	X	X	X	X	X	X	X	X	X	X		X	X	X	X
Is it stated who the target population the measurement tool was developed for?	X	X	X	X	X		X		X		X	X	X	X	X		X	X	X	X	X	X	X
**B. Cognitive interview/pilot test (Comprehensiveness, Comprehensibility)**																							
• Was the cognitive interview study or other pilot test performed in a sample representing the target population?		X		X					X					X				X			X		
• Were patients asked about the comprehensibility of the measurement tool?									X				X					X					
• Were patients asked about the comprehensiveness of the measurement tool?		X											X					X					

*Note*: Data were extracted by the third and fourth authors between August and September 2023, and following the update in March 2024.

## Discussion

This systematic review aimed to identify self-regulation measures applicable to infants, children, and young people aged 0 to 18 years, identifying 23 measures from 48 studies. The findings reveal significant gaps, particularly the scarcity of measures suitable for young children. Additionally, the study highlights that most measures have been insufficiently evaluated for psychometric properties. Overall, the findings provide a comprehensive overview of currently available measures, offering valuable insights for clinicians in selecting appropriate self-regulation assessments and recognising the limitations of existing tools.

One of the main findings concerns the availability of measures for different age cohorts. First, there is a significant scarcity of measures suitable for young children, which presents clinical challenges in early intervention, especially during the critical period when addressing self-regulation difficulties is paramount [77]. This scarcity underscores the need for well-developed and ecologically valid measures to assess self-regulation in preschool-age children, given its predictive nature for social and academic skills throughout childhood [78]. In addition, the majority of measures identified are self-report, particularly prevalent among adolescents aged 11–17 years. While self-report measures offer time efficiency for clinicians, their suitability may be limited for younger or neurodiverse children who are still developing their self-awareness and the ability to process, understand and communicate their emotions and experiences [79].

Another significant finding underscores the insufficient examination of psychometric properties across measures, with most studies only including 1–2 evaluations per measure. In addition, the majority of investigations were conducted by the developers of measures, resulting in a lack of independent evaluations. In contrast to other measures, the ERC was the most extensively evaluated, with nine studies exploring various psychometric properties. Given that the median age of children reported in a recent systematic review of self-regulation interventions was six years [80], the ERC emerges as the most researched measure for clinicians working with children aged 3–12 years who commonly require intervention for self-regulation. For clinicians supporting older children and young people, the DERS and its brief version (11–17 years) and ESCQ (12–17 years) were the most extensively researched measures (both self-report), each with four or more studies evaluating psychometric properties.

Of note, measurement error was not evaluated for any of the 23 measures, which raises concerns given its importance in reliability assessment. Measurement error estimates systematic and random errors in a client’s score that are not attributed to true changes in the measured construct [23]. This is particularly crucial when assessing pre- to post-intervention change.

However, most measures had been assessed for validity, including structural validity, hypothesis testing, internal consistency, and cross-cultural validity. Notably, content validity was inadequately assessed for most measures despite its significance in ensuring that the measure adequately reflects the construct of interest. Content validity involves both item generation and cognitive interview/pilot testing to ensure relevance, comprehensiveness and comprehensibility [15]. While many measures included the construct’s definition and conceptual framework, they fell short in evaluating comprehensiveness and comprehensibility. This is particularly concerning, given the prevalence of self-report measures. Although these measures allow individuals to self-report against included items, very few measures conducted cognitive interviews or other pilot tests on a sample representing the target population or sought feedback on the comprehensibility or comprehensiveness of the measurement tools. This approach hampers the understanding and interpretation of items by respondents, which may lead to capturing data that does not encapsulate the construct of self-regulation. Finally, although the included measures evaluated all three domains of self-regulation, most measures had a particular focus on emotional regulation, which is reflected in the names of many measures.

### Limitations

While aiming for the rigour of this systematic review, several limitations should be considered when interpreting the results. Firstly, due to the absence of a universally agreed-upon definition for the construct of self-regulation and its domains, we relied on each domain’s shared understanding to review each measure’s items and determine the measures for inclusion in our review. Secondly, we assessed whether each type of psychometric property was examined for each measure but did not rate the quality of the psychometric properties of the reported information about the instruments. Future studies should utilise the COSMIN risk of bias [23] to evaluate the methodological quality of each psychometric article and the quality of the various psychometric properties to guide assessment and treatment planning decisions. Further, assessing responsiveness as a psychometric property was beyond the scope of this systematic review, as it would require a different search strategy. Subsequent studies could explore whether the measures included in this review have been employed as outcome measures and evaluate their responsiveness to changes in self-regulation. Lastly, this review only included full-text English studies, potentially overlooking measures developed in languages other than English.

## Conclusion

This systematic review identified 48 studies that reported evidence of the psychometric properties of 23 self-regulation measures used with infants, children, and young people aged 0 to 18 years. Most measures had limited investigation of psychometric properties, except the ERC, the most extensively evaluated measure. Despite the critical role of self-regulation in the clinical assessment and treatment of children, this review underscores the need for more research examining the psychometric properties of self-regulation measures used with children. Directions for future research can be considered in terms of the continued evaluation of existing measures or the development of new measures. Notably, one avenue for future research is the development of measures for young children, particularly those aged three years and younger. Such measures should consider parent and teacher reports as well as observational assessments across home and other care contexts. Another avenue for further research is evaluating measurement errors in existing measures. Additionally, more parent- and teacher-report and observational assessments are needed to evaluate self-regulation measures for older children and young people.

## Supporting information

S1 File Search terms and strategies. (PDF)

S2 File PRISMA checklist. (PDF)

S3 File List of identified studies for the full-text review. (PDF)

S4 File Quality assessment of includqed studies. (PDF)
