# Supplementary material for: A systematic review of self-regulation measures in children: Exploring characteristics and psychometric properties
Source: PLoS One. 2024 Sep 19;19(9):e0309895. doi: 10.1371/journal.pone.0309895 (PMC11412528; doi:10.1371/journal.pone.0309895)
Supplement: S1 File — (PDF) [file pone.0309895.s001.pdf]

Search terms & strategies

**EMBASE**<br><br>((psychometry/ or validity/ or reliability/ or measurement error/ or measurement precision/ or measurement repeatability/ or error/ or statistical bias/ or test retest reliability/ or intrarater reliability/ or interrater reliability/ or accuracy/ or criterion validity/ or internal validity/ or face validity/ or external validity/ or discriminant validity/ or concurrent validity/ or qualitative validity/ or construct validity/ or content validity/) OR (Psychometric* or reliability or validit* or reproducibility or bias)) AND ((child/ or adolescent/ or infant/) OR (teen* or student* or young people or young person*)) AND ((self report/ or questionnaire/ or clincal assessment tool/) OR (measure* or parent report* or observation* or tool* or assessment* or instrument* or scales* or test* or physiologic* effect* or physiologic* respon* or emotional respon* or survey*)) AND ((emotionality/) OR (emotion* regulation.ti,ab. OR behavior* regulation.ti,ab. OR behaviour* regulation.ti,ab. OR self regulat*.ti,ab. OR emotion* manag*.ti,ab. OR affect regulation.ti,ab. OR emotion* competenc*.ti,ab. OR effortful control.ti,ab. OR behavior* manag*.ti,ab. OR behaviour* manag*.ti,ab. OR emotion* dysregulation.ti,ab. OR emotion* dysregulation.ti,ab.))
**MEDLINE**<br><br>(Psychometrics/ or Reproducibility Of Results/) AND ((Infant/ or Child/ or Pediatrics/ or Adolescent/) OR (teen* or young people or young person*)) AND ((Surveys and Questionnaires/ or Outcome Assessment (Health Care)/ or Neuropsychological Tests/ or Psychological Tests/ or Self Report/ or Observation/) OR (parent report or tool* or assessment* or scales or test*)) AND ((Self-Control/ or Child Behavior Disorders/ or Disruptive, Impulse Control, and Conduct Disorders/) OR ((emotion* regulation or behavior* regulation or behaviour* regulation or self regulat* or emotion* manag* or affect regulation or emotion* competenc* or effortful control or behavior* manag* or behaviour* manag* or emotion* dysregulation or emotion* disregulation).ti,ab.)))
**PsycINFO**<br><br>((DE "Psychometrics" OR DE "Statistical Validity" OR DE "Test Validity" OR DE "Statistical Reliability" OR DE "Test Reliability" OR DE "Error of Measurement" OR DE "Errors" OR DE "Response Bias" OR DE "Interrater Reliability" OR DE "Repeated Measures") OR (Psychometric* or reliability or validit* or reproducibility or bias)) AND ((DE “Pediatrics”) OR (child* or adolescent* or infant* student* or teen* or young people or young person*) AND ((DE “Emotional State Measures” OR DE “Emotional Assessment”) OR (measure* or self report* or parent report* or observation* or tool* or assessment* or instrument* or scales* or test* or questionnaire* or physiologic* effect* or physiologic* respon* or emotional* respon* or survey*)) AND ((DE “Emotion* Regulation” OR DE “Emotional Control” OR DE “Self Regulation” OR DE “Self Control” OR DE “ Emotional Stability” OR DE “Emotional Responses” OR DE “Emotional Disturbances”) OR (TI “emotion* regulation” OR AB “emotion* regulation” OR TI “behavior* regulation” OR AB “behavior regulation” OR TI “behaviour regulation” OR AB “behaviour regulation” OR TI “self regulat*” OR AB “self regulat*” OR TI “emotion* manag*” OR AB “emotion* manag*” OR TI “affect regulation” OR AB “affect regulation” OR TI “emotion* competenc*” OR AB “emotion* competenc*” OR TI “effortful control” OR AB “effortful control” OR TI “behavior* manag*” OR AB “behavior* manag*” OR TI “behaviour* manag*” OR AB “behaviour* manag*” OR TI “emotion* dysregulation” OR AB “emotion* dysregulation” OR TI “emotion* disregulation” OR AB “emotion* disregulation”))
**Scopus**<br><br>(TITLE-ABS-KEY (pediatric* OR child* OR infant* OR adolescent* OR teen* OR "young person" OR "young people*" OR student*)) AND (TITLE-ABS-KEY (psychometric* OR

psychometry OR validit* OR reproducibility OR reliability OR "measurement error" OR "measurement precision" OR "measurement repeatability" OR error OR bias OR accuracy)) AND (TITLE-ABS-KEY (measure* OR "self report*" OR "parent report*" OR observation* OR tool* OR assessment* OR instrument* OR scales* OR test* OR questionnaire* OR "physiologic* effect*" OR "physiologic* respon*" OR "emotional respon*" OR survey*)) AND (TITLE-ABS-KEY ("emotion* regulation" OR "behavior* regulat*" OR "behaviour* regulat*" OR "self regulat*" OR "emotion* manag*" OR "affect regulation*" OR "emotion* competenc*" OR "effortful control" OR "behaviour* manag*" OR "behavior* manag*" OR "emotion* dysregulation")) AND (LIMIT-TO (LANGUAGE, "English"))
**CINAHL**<br><br>((MH "Psychometrics") OR (MH "Measurement Issues and Assessments") OR (MH "Validity") OR (MH "Predictive Validity") OR (MH "Reliability and Validity") OR (MH "Internal Validity") OR (MH "Face Validity") OR (MH "External Validity") OR (MH "Discriminant Validity") OR (MH "Criterion-Related Validity") OR (MH "Consensual Validity") OR (MH "Concurrent Validity") OR (MH "Qualitative Validity") OR (MH "Construct Validity") OR (MH "Content Validity") OR (MH "Instrument Validation") OR (MH "Validation Studies") OR (MH "Test-Retest Reliability") OR (MH "Sensitivity and Specificity") OR (MH "Reproducibility of Results") OR (MH "Reliability") OR (MH "Intrarater Reliability") OR (MH "Interrater Reliability") OR (MH "Measurement Error") OR (MH "Bias (Research)") OR (MH "Selection Bias") OR (MH "Sampling Bias") OR (MH "Precision") OR (MH "Sample Size Determination") OR (MH "Repeated Measures") OR (Psychometric* or reliability or validit* or reproducibility or bias)) AND ((MH “Child+”) OR (MH “Adolescence”) OR (MH “Infant+”) OR (teen* or student* or young people or young person*)) AND ((MH “Self Report+”) OR (MH “Clinical Assessment Tools+”) OR (MH “Outcome Assessment”) OR (MH “Scales”) OR (MH “Questionnaires”) OR OR (MH “Surveys+”) OR (MH “Biophysical Instruments+”) OR (MH “Self Assessment”) OR (MH “Research Instruments+”) OR (measure* or parent report* or observation* or tool* or assessment* or instrument* or test* or physiologic* effect* or physiologic* respon* or emotional* respon*)) AND ((MH “Self Regulation”) OR (TI “emotion* regulation” OR AB “emotion* regulation” OR TI “behavior* regulation” OR AB “behavior* regulation” OR TI “behaviour* regulation” OR AB “behaviour* regulation” OR TI “self regulat*” OR AB “self regulat*” OR TI “emotion* manag*” OR AB “emotion* manag*” OR TI “affect regulation” OR AB “affect regulation” OR TI “emotion* competenc” OR AB “emotion* competenc” OR TI “effortful control” OR AB “effortful control” OR TI “behavior* manag*” OR AB “behavior* manag* OR TI “behaviour* manag*” OR AB “behaviour* manag*” OR TI “emotion* dysregulation” OR AB “emotion* dysregulation” OR TI “emotion* disregulation” OR AB “emotion* disregulation”))
**ERIC**<br><br>((DE "Psychometrics" OR DE "Validity" OR DE "Reliability" OR DE "Error of Measurement" OR DE "Bias" OR DE "Interrater Reliability" OR DE "Accuracy" OR DE "Predictive Validity" OR DE "Construct Validity" OR DE "Content Validity") OR (Psychometric* or reliability or validit* or reproducibility or bias)) AND ((DE “Children” OR DE “Adolescents” OR DE “Infants” OR DE “Students”) OR (teen* or young people or young person*)) AND ((DE “Measures (Individuals)” OR DE “Affective Measures”) OR (measure* or self report* or parent report* or observation* or tool* or assessment* or instrument* or scales* or test* or questionnaire* or physiologic* effect* or physiologic* respon* or emotional* respon* or survey*)) AND ((DE “Emotional Adjustment” OR DE “Self Control” OR DE “Affective Behavior”) OR (TI “emotion* regulation” OR AB “emotion* regulation” OR TI “behavior* regulation” OR AB “behavior regulation” OR TI “behaviour regulation” OR AB “behaviour regulation” OR TI “self regulat*” OR AB “self regulat*” OR TI “emotion* manag*” OR AB “emotion* manag*” OR TI “affect regulation” OR AB “affect regulation” OR TI “emotion* competenc*” OR AB “emotion* competenc*” OR TI “effortful

control” OR AB “effortful control” OR TI “behavior* manag*” OR AB “behavior* manag*” OR TI “behaviour* manag*” OR AB “behaviour* manag*” OR TI “emotion* dysregulation” OR AB “emotion* dysregulation” OR TI “emotion* disregulation” OR AB “emotion* disregulation”))
