# Supplementary material for: A systematic review of self-regulation measures in children: Exploring characteristics and psychometric properties
Source: PLoS One. 2024 Sep 19;19(9):e0309895. doi: 10.1371/journal.pone.0309895 (PMC11412528; doi:10.1371/journal.pone.0309895)
Supplement: S3 File — (PDF) [file pone.0309895.s003.pdf]

List of identified studies for the full-text review

	Article	Inclusion/exclusion
1	Abdi, S., Taban, S., & Ghaemian, A. (2012). *Cognitive emotion regulation questionnaire: Validity and reliability of the persian translation of the CERQ (36-item).* Paper presented at the Procedia - Social and Behavioral Sciences.	Does not contain all three domains of SR
2	Allan, N. P., Lonigan, C. J., & Wilson, S. B. (2013). Psychometric evaluation of the Children's Behavior Questionnaire-Very Short Form in preschool children using parent and teacher report. *Early Childhood Research Quarterly, 28* (2), 302-313.	Does not contain all three domains of SR
3	Alonso-Tapia, J., Caleron, E. P., & Ruiz, M. A. (2014). Development and validity of the Emotion and Motivation Self-Regulation Questionnaire (EMSR-Q). *The Spanish Journal of Psychology Vol 17, 2014, ArtID E55, 17* .	Does not contain all three domains of SR
4	Anunciação, L., Chen, C. Y., Pereira, D. A., & Landeira-Fernandez, J. (2019). Factor structure of a social-emotional screening instrument for preschool children. Psico-USF, 24, 449-461.	Criteria not met
5	Aslan, A. E., & Sevincler-Togan, S. (2009). A Service for emotion management: Turkish Version of the Adolescent Anger Rating Scale (AARS). *Educational Sciences: Theory and Practice, 9* (2), 391-400.	Included
6	Aurora-Adina, I. (2011). Assessing the social-emotional skills in Romanian teenagers. Procedia-Social and Behavioral Sciences, 30, 876-882.	Articles/assessment not available in English
7	Backer-Grøndahl, A., Nærde, A., Ulleberg, P., & Janson, H. (2016). Measuring Effortful Control Using the Children's Behavior Questionnaire-Very Short Form: Modeling Matters. *Journal of Personality Assessment, 98* (1), 100-109.	Does not contain all three domains of SR
8	Baggett, K. M., Davis, B., Feil, E. G., Sheeber, L. L., Landry, S. H., Carta, J. J., & Leve, C. (2010). Technologies for expanding the reach of evidence-based interventions: Preliminary results for promoting social-emotional development in early childhood. *Topics in Early Childhood Special Education, 29* (4), 226-238. doi:10.1177/0271121409354782	Included
9	Bar-Haim, Y., Bar-Av, G., & Sadeh, A. (2011). Measuring children's regulation of emotion-expressive behavior. *Emotion, 11* (2), 215-223.	Does not contain all three domains of SR
10	Barańczuk, U., Zawadzki, B., Kamholz, B. W., Hayes, A. M., & Lawrence, A. (2017). Psychometric properties of a Polish version of the Inventory of Cognitive Affect Regulation Strategies (ICARUS). Psychological Assessment, 29(3), e1-e12.	Criteria not met
11	Bartroli, M., Angulo-Brunet, A., Bosque-Prous, M., Clotas, C., & Espelt, A. (2022). The Emotional Competence Assessment Questionnaire (ECAQ) for children aged from 3 to 5 years: validity and reliability evidence. *Education Sciences, 12* (7), 489. doi:10.3390/educsci12070489	Included
12	Barve, C. (2004). Narrative assessment of emotion regulation in adolescent suicide attempters. The Catholic University of America.	Dissertation
13	Baurain, C., & Nader-Grosbois, N. (2011). Validation of a method of assessing socioemotional regulation in preschoolers. European Review of Applied Psychology, 61(4), 185-194.	Criteria not met

14	Betegón, E., Rodríguez-Medina, J., Del-Valle, M., & Irurtia, M. J. (2022). Emotion Regulation in Adolescents: Evidence of the Validity and Factor Structure of the Cognitive Emotion Regulation Questionnaire (CERQ). *International Journal of Environmental Research and Public Health, 19* (6).	Does not contain all three domains of SR
15	Bilek, E. L. (2015). A multimethod assessment of emotion regulation and associations with anxiety symptoms in a clinical sample of school-age youth. University of Miami.	Dissertation
16	Bowie, B. H. (2010). Emotion regulation related to children's future externalizing and internalizing behaviors. *Journal of Child & Adolescent Psychiatric Nursing, 23* (2), 74-83. doi:10.1111/j.1744-6171.2010.00226.x	Included
17	Boyer, W. (2023). Development, Construct Validation, and Normalization of a New Early Childhood Self-Regulation Assessment Scale. *Early Childhood Education Journal, 51* (4), 627-640.	Does not contain all three domains of SR
18	Bunford, N., Dawson, A. E., Evans, S. W., Ray, A. R., Langberg, J. M., Owens, J. S., . . . Allan, D. M. (2020). The Difficulties in Emotion Regulation Scale–Parent Report: A psychometric investigation examining adolescents with and without ADHD. *Assessment, 27* (5), 921-940. doi:10.1177/1073191118792307	Included
19	Burney, D. M., & Kromrey, J. (2001). Initial development and score validation of the Adolescent Anger Rating Scale. *Educational and Psychological Measurement, 61* (3), 446-460. doi:10.1177/00131640121971310	Included
20	Burton, A. L., Brown, R., & Abbott, M. J. (2022). Overcoming difficulties in measuring emotional regulation: Assessing and comparing the psychometric properties of the DERS long and short forms. Cogent Psychology, 9(1), 2060629.	Mean age not <18
21	Carey, K. B., Neal, D. J., & Collins, S. E. (2004). A psychometric analysis of the self-regulation questionnaire. *Addictive Behaviors, 29* (2), 253-260.	Does not contain all three domains of SR
22	Carroll, A., Hemingway, F., Ashman, A., & Bower, J. (2012). Establishing the Psychometric Properties of an Interactive, Self-Regulation Assessment Battery for Young Offenders. *Australian Journal of Guidance and Counselling, 22* (1), 102-121.	Does not contain all three domains of SR
23	Cerolini, S., Zagaria, A., Vacca, M., Spinhoven, P., Violani, C., & Lombardo, C. (2022). Cognitive Emotion Regulation Questionnaire—Short: Reliability, Validity, and Measurement Invariance of the Italian Version. *Behavioral Sciences, 12* (12).	Does not contain all three domains of SR
24	Chamizo-Nieto, M. T., Rey, L., & Sánchez-álvarez, N. (2020). Validation of the Spanish version of the cognitive emotion regulation questionnaire in adolescents. *Psicothema, 32* (1), 153-159.	Does not contain all three domains of SR
25	Charak, R., Byllesby, B. M., Fowler, J. C., Sharp, C., Elhai, J. D., & Frueh, B. C. (2019). Assessment of the revised Difficulties in Emotion Regulation Scales among adolescents and adults with severe mental illness. *Psychiatry research, 279* , 278-283. doi:10.1016/j.psychres.2019.04.010	Included
26	Chen, C. Y. (2017). Examination of Psychometric Properties of a Translated Social-Emotional Screening Test: The Taiwanese Version of the Ages and Stages Questionnaires: Social-Emotional. University of Oregon.	Criteria not met

27	Chen, C. Y., Filgueiras, A., Squires, J., & Landeira-Fernandez, J. (2016). Examining the factor structure of an early childhood social emotional screening assessment. Journal of Special Education and Rehabilitation, 17(3-4), 89-104.	Criteria not met
28	Chen, W., Zhang, G., Tian, X., & Wang, L. (2023). Psychometric properties and measurement invariance of the emotion regulation questionnaire in Chinese left-behind children. *Current Psychology, 42* (11), 8833-8843.	Does not contain all three domains of SR
29	Chen, Y. H., & Lin, Y. J. (2018). Validation of the short self-regulation questionnaire for Taiwanese college students (TSSRQ). *Frontiers in Psychology, 9* (MAR).	Does not contain all three domains of SR
30	Chen, Y.-H., & Lin, Y.-J. (2020). Revalidating the Taiwanese Self-Regulation Questionnaire (New TSSRQ) and exploring its relationship with college students' psychological well-being. *Frontiers in Psychology Vol 11, 2020, ArtID 1192.*	Does not contain all three domains of SR
31	Chen, Y., Liu, W., & Zhang, X. (2016). Chinese revision of the emotion regulation questionnaire for children and adolescents. *Chinese Journal of Clinical Psychology, 24* (2), 259-263.	Does not contain all three domains of SR
32	Chen, Y., Wang, S., & Ling, Y. (2023). Measurement Invariance of the Emotion Regulation Questionnaire for Children and Adolescents in Chinese and American Adolescents Samples. *Journal of Psychoeducational Assessment, 41* (1), 88-99.	Does not contain all three domains of SR
33	Chennaz, L., Valente, D., Baltenneck, N., Baudouin, J.-Y., & Gentaz, E. (2022). Emotion regulation in blind and visually impaired children aged 3 to 12 years assessed by a parental questionnaire. *Acta psychologica, 225* , 103553. doi:10.1016/j.actpsy.2022.103553	Included
34	Cho, M.-H. and Y. Cho (2017). "Self-Regulation in Three Types of Online Interaction: A Scale Development." Distance Education 38(1): 70-83.	Mean age not <18
35	Ciucci, E., Baroncelli, A., Grazzani, I., Ornaghi, V., & Caprin, C. (2016). Emotional arousal and regulation: Further evidence of the validity of the “How I Feel” Questionnaire for use with school-age children. Journal of School Health, 86(3), 195-203.	Does not contain all three domains of SR
36	Clark, D., Listro, C. J., Lo, S. L., Durbin, C., Donnellan, M., & Neppl, T. K. (2016). Measurement invariance and child temperament: An evaluation of sex and informant differences on the Child Behavior Questionnaire. *Psychological Assessment, 28* (12), 1646-1662.	Does not contain all three domains of SR
37	Cleary, T. J. (2006). The Development and Validation of the Self-Regulation Strategy Inventory--Self-Report. *Journal of School Psychology, 44* (4), 307-322.	Does not contain all three domains of SR
38	Cleary, T. J., Dembitzer, L., & Kettler, R. J. (2015). Internal Factor Structure and Convergent Validity Evidence: The Self-Report Version of Self-Regulation Strategy Inventory. *Psychology in the Schools, 52* (9), 829-844.	Does not contain all three domains of SR
39	Coelho, V. A., Sousa, V., & Marchante, M. (2016). Social and emotional competencies evaluation questionnaire—Teacher’s version: validation of a short form. Psychological Reports, 119(1), 221-236.	Criteria not met
40	Conesa, P. J., & Duñabeitia, J. A. (2022). Adaptation and Validation to Spanish elementary school children of the Academic Self-Regulation Questionnaire (SRQ-A). *Electronic Journal of Research in Educational Psychology, 20* (57), 403-426.	Does not contain all three domains of SR

41	Conesa, P., & Duñabeitia, J. A. (2022). Adaptation and Validation to Spanish elementary school children of the Academic Self-Regulation Questionnaire (SRQ-A). Electronic Journal of Research in Education Psychology, 20(57), 403-426.	Criteria not met
42	Corral-Frías, N. S., Veldardez Soto, S. N., Camacho Amay, M. Y., & McRae, K. (2022). Validity of Emotion Regulation Questionnaire in Diverse Mexican Samples Using Two Different Spanish Translations. *Interamerican Journal of Psychology, 56* (3).	Does not contain all three domains of SR
43	Costa, A., Faria, L., & Takšić, V. (2016). Cross-cultural invariance of emotional skills and competence questionnaire between Portugal and Croatia. *The Spanish Journal of Psychology, 19* , E31. doi:10.1017/sjp.2016.33	Included
44	Cracco, E., Van Durme, K., & Braet, C. (2015). Validation of the FEEL-KJ: an instrument to measure emotion regulation strategies in children and adolescents. PloS one, 10(9), e0137080.	Criteria not met
45	d'Acremont, M., & Van der Linden, M. (2007). How is impulsivity related to depression in adolescence? Evidence from a French validation of the cognitive emotion regulation questionnaire. *Journal of Adolescence, 30* (2), 271-282.	Does not contain all three domains of SR
46	Daneri, M. P., Sulik, M. J., Raver, C. C., & Morris, P. A. (2018). Observers' reports of self-regulation: Measurement invariance across sex, low-income status, and race/ethnicity. *Journal of Applied Developmental Psychology, 55* , 14-23. doi:10.1016/j.appdev.2017.02.001	Included
47	Danisman, S., Dereli, I. E., Akin, D. Z., & Yaya, D. (2016). Examining the psychometric properties of the Emotional Regulation Checklist in 4- and 5-year-old preschoolers. *Electronic Journal of Research in Educational Psychology, 14* (3), 534-556. doi:10.14204/ejrep.40.15124	Included
48	Day, T. N., Mazefsky, C. A., Yu, L., Zeglen, K. N., Neece, C. L., & Pilkonis, P. A. (2024). The Emotion Dysregulation Inventory−Young Child: Psychometric Properties and Item Response Theory Calibration in 2- to 5-Year-Olds. *Journal of the American Academy of Child and Adolescent Psychiatry, 63* (1), 52-64.	Does not contain all three domains of SR
49	de la Osa, N., Granero, R., Penelo, E., Domènech, J. M., & Ezpeleta, L. (2014). The Short and Very Short Forms of the Children’s Behavior Questionnaire in a Community Sample of Preschoolers. *Assessment, 21* (4), 463-476.	Does not contain all three domains of SR
50	Demirpence Secinti, D., & Sen, E. (2023). Reliability and validity of the brief version of the difficulties in emotion regulation scale in a sample of Turkish adolescents. *BMC psychology, 11* (1), 165. doi:10.1186/s40359-023-01199-y	Included
51	Denham, S. A., Bassett, H. H., Zinsser, K. M., Bradburn, I. S., Bailey, C. S., Shewark, E. A., ... & Kianpour, S. (2020). Computerized social-emotional assessment measures for early childhood settings. Early Childhood Research Quarterly, 51, 55-66.	Criteria not met
52	Di Giunta, L., Iselin, A.-M. R., Eisenberg, N., Pastorelli, C., Gerbino, M., Lansford, J. E., . . . Thartori, E. (2017). Measurement Invariance and Convergent Validity of Anger and Sadness Self-Regulation Among Youth From Six Cultural Groups. *Assessment, 24* (4), 484-502.	Does not contain all three domains of SR
53	Dias, P. C., del Castillo, J. A. G., & Moilanen, K. L. (2014). The adolescent self-regulatory inventory (ASRI) adaptation to Portuguese context. *Paidéia (Ribeirão Preto), 24* , 155-164.	Included

54	Ding, F., Wang, X., Cheng, C., He, J., Zhao, H., Wu, D., & Yao, S. (2021). Psychometric Properties and Measurement Invariance of the Cognitive Emotion Regulation Questionnaire in Chinese Adolescents With and Without Major Depressive Disorder: A Horizontal and Longitudinal Perspective. *Frontiers in Psychiatry, 12* . doi:10.3389/fpsyt.2021.736887	Does not contain all three domains of SR
55	Dinovo Jr, S. A. (2009). A Multimethod Assessment of Effortful Self-Regulation in Personality Research: Temperamental, Neuropsychological, and Psychophysiological Concomitants. The Ohio State University.	Dissertation
56	Domínguez-Sánchez, F. J., Lasa-Aristu, A., Amor, P. J., & Holgado-Tello, F. P. (2013). Psychometric Properties of the Spanish Version of the Cognitive Emotion Regulation Questionnaire. *Assessment, 20* (2), 253-261.	Does not contain all three domains of SR
57	Enebrink, P., Björnsdotter, A., & Ghaderi, A. (2013). The emotion regulation questionnaire: Psychometric properties and norms for Swedish parents of children aged 10-13 years. *Europe's Journal of Psychology, 9* (2), 289-303.	Does not contain all three domains of SR
58	Erreygers, S., & Spooren, P. (2017). Factor structure of the affective style questionnaire in flemish adolescents. *Psychologica Belgica, 57* (2), 112-122.	Does not contain all three domains of SR
59	Ettel, D. J. (2009). The measurement of emotion regulation: A confirmatory analysis. University of Oregon.	Dissertation
60	Eva, D., & Denes, K. (2019). Hungarian version of the Children's Behavior Questionnaire very short form (CBQ VS). *Mentalhigiene es Pszichoszomatika, 20* (2), 159-179.	Does not contain all three domains of SR
61	Faria, L., & Lima-Santos, N. (2012). Emotional intelligence in the Portuguese academic context: Validation studies of "the Emotional Skills and Competence Questionnaire" (ESCQ). *Behavioral Psychology/Psicologia Conductual, 20* (1), 91-102.	Included
62	Faria, L., Lima Santos, N., Takšic, V., Räty, H., Molander, B., Holmström, S., . . . Fernández-Berrocal, P. (2006). Cross-cultural validation of the Emotional Skills and Competence Questionnaire (ESCQ). *Psicologia, 20* (2), 95-127.	Included
63	Fernandes, V. R., Becker, D. R., McClelland, M. M., & Deslandes, A. C. (2023). Head-Toes-Knees-Shoulders task and EF in two samples of adolescents in Brazil and United States. *Frontiers in Psychology, 14* .	Does not contain all three domains of SR
64	Fombouchet, Y., Lannegrand, L., & Lucenet, J. (2023). The Contextualized Emotion Regulation Survey for Adolescents (CERSA): How does emotion regulation vary according to context?. British journal of developmental psychology, 41(3), 306-323.	Articles/assessment not available in English
65	Frohn, S. R. (2018). An evaluation and revision of the Children's Behavior Questionnaire Effortful Control Scales.DP - 2018. *Dissertation Abstracts International: Section B: The Sciences and Engineering, 79* (1-B(E))	Does not contain all three domains of SR
66	Gajda, M., Małkowska-Szkutnik, A., & Rodzeń, W. (2022). Self-regulation in adolescents: Polish adaptation and validation of the Self-Regulation Scale. *International journal of environmental research and public health, 19* (12), 7432. doi:10.3390/ijerph19127432	Included
67	Gaspar, T., Tomé, G. Q., Simões, C., & de Matos, M. G. (2015). Self-Regulatory Strategies in Pre-Adolescents and Adolescents: Portuguese Version of TESQ-E. Psicologia, Reflexão e Crítica, 28(4), 649.	Articles/assessment not available in English

68	Georg, S., Genser, B., Fischer, J., Sachse, S., & De Bock, F. (2023). Development and validation of a self-regulation scale within the German version of the Early Development Instrument. BMC pediatrics, 23(1), 509.	Does not contain all three domains of SR
69	Gerson, A. C., Gerring, J. P., Freund, L., Joshi, P. T., Capozzoli, J., Brady, K., & Denckla, M. B. (1996). The Children's Affective Lability Scale: A psychometric evaluation of reliability. *Psychiatry Research, 65* (3), 189-198.	Does not contain all three domains of SR
70	Giromini, L., et al. (2012). "Cultural Adaptation of the Difficulties in Emotion Regulation Scale: Reliability and Validity of an Italian Version." Journal of Clinical Psychology 68(9): 989-1007.	Mean age not <18
71	Giuliani, M. F., Villar, F., Arias, C. J., & Serrat, R. (2015). Development and structural validation of a scale to assess regulation of anger and sadness in interpersonal situations. *Anuario de Psicologia, 45* (1), 115-130.	Does not contain all three domains of SR
72	Giunta, L., Iselin, A. M. R., Eisenberg, N., Pastorelli, C., Gerbino, M., Lansford, J. E., . . . Thartori, E. (2017). Measurement Invariance and Convergent Validity of Anger and Sadness Self-Regulation Among Youth From Six Cultural Groups. *Assessment, 24* (4), 484-502.	Does not contain all three domains of SR
73	Gomes, M., Monteiro, V., Mata, L., Peixoto, F., Santos, N., & Sanches, C. (2019). The Academic Self-Regulation Questionnaire: a study with Portuguese elementary school children. *Psicologia: Reflexao e Critica, 32* (1).	Does not contain all three domains of SR
74	Gómez-Ortiz, O., Romera, E. M., Ortega-Ruiz, R., Cabello, R., & Fernández-Berrocal, P. (2016). Analysis of emotion regulation in spanish adolescents: Validation of the emotion regulation questionnaire. *Frontiers in Psychology, 6* (JAN).	Does not contain all three domains of SR
75	Gong, J., Wang, M.-C., Zhang, X., Zeng, H., & Yang, W. (2022). The Emotion Regulation Questionnaire for Children and Adolescents (ERQ-CA): Factor structure and measurement invariance in a Chinese student samples. *Journal of Personality Assessment, 104* (6), 774-783.	Does not contain all three domains of SR
76	Gonzales, C. R., Bowles, R., Geldhof, G., Cameron, C. E., Tracy, A., & McClelland, M. M. (2021). The Head-Toes-Knees-Shoulders Revised (HTKS-R): Development and psychometric properties of a revision to reduce floor effects. *Early Childhood Research Quarterly, 56* , 320-332.	Does not contain all three domains of SR
77	Gonzalez Rodriguez, C. J. (1998). The child behavior questionnaire: a useless tool. *Atencion Primaria, 22* (1), 60-63.	Does not contain all three domains of SR
78	Gordeeva, T. O., Sychev, O. A., & Lynch, M. F. (2020). The construct validity of the russian version of the modified academic self-regulation questionnaire (SRQ-A) among elementary and middle school children. *Psychology in Russia: State of the Art, 13* (3), 16-34.	Does not contain all three domains of SR
79	Gosling, C. J., Noblecourt, K., & Moutier, S. (2018). French version of the emotion regulation questionnaire for children and adolescents. *Enfance, 2018* (2), 291-303.	Does not contain all three domains of SR
80	Gouveia, V. V., de Moura, H. M., de Oliveira, I. C. V., Ribeiro, M. G. C., Rezende, A. T., & Brito, T. R. S. (2018). Emotional Regulation Questionnaire (ERQ): Evidence of Construct Validity and Internal Consistency. *Psico-USF, 23* (3), 461-471	Does not contain all three domains of SR
81	Gračanin, A., Kardum, I., & Gross, J. J. (2020). The Croatian version of the Emotion Regulation Questionnaire: Links with higher- and lower-level personality traits and mood. *International Journal of Psychology, 55* (4), 609-617.	Does not contain all three domains of SR

82	Graser, J., Bohn, C., Kelava, A., Schreiber, F., Hofmann, S. G., & Stangier, U. (2012). The "affective style questionnaire (ASQ)": German adaptation and validity. *Diagnostica, 58* (2), 100-111.	Does not contain all three domains of SR
83	Graser, J., Heimlich, C., Kelava, A., Hofmann, S. G., Stangier, U., & Schreiber, F. (2019). Assessment of emotion regulation in adolescents with the Affective Style Questionnaire-Youth" ASQ-Y. *Diagnostica, 65* (1), 49-59.	Does not contain all three domains of SR
84	Gratz, K. L., & Roemer, L. (2004). Multidimensional assessment of emotion regulation and dysregulation: Development, factor structure, and initial validation of the difficulties in emotion regulation scale. Journal of psychopathology and behavioral assessment, 26, 41-54.	Mean age not <18
85	Gullone, E., & Taffe, J. (2012). The emotion regulation questionnaire for children and adolescents (ERQ-CA): A psychometric evaluation. *Psychological Assessment, 24* (2), 409-417.	Does not contain all three domains of SR
86	Gunzenhauser, C., von Suchodoletz, A., & McClelland, M. M. (2017). Measuring cognitive reappraisal and expressive suppression in children: A parent-rating version of the emotion regulation questionnaire. *European Journal of Developmental Psychology, 14* (4), 489-497.	Does not contain all three domains of SR
87	Haag, A.-C., Cha, C. B., Noll, J. G., Gee, D. G., Shenk, C. E., Schreier, H. M., . . . Bonanno, G. A. (2023). The Flexible Regulation of Emotional Expression Scale for Youth (FREE-Y): Adaptation and validation across a varied sample of children and adolescents. *Assessment, 30* (4), 1265-1284.	Does not contain all three domains of SR
88	Hagstrøm, J., Spang, K. S., Christiansen, B. M., Maigaard, K., Vangkilde, S., Esbjørn, B. H., . . . Plessen, K. J. (2019). The Puzzle of Emotion Regulation: Development and Evaluation of the Tangram Emotion Coding Manual for Children. *Frontiers in Psychiatry, 10* .	Does not contain all three domains of SR
89	Hasani, J. (2017). Validity, reliability and confirmatory factor struture of adolescent's Emotion Regulation Questionnaire. *Developmental Psychology: Journal of Iranian Psychologists, 13* (51), 285-295.	Does not contain all three domains of SR
90	Hasani, J., Emadi Chashmi, S. J., Zakiniaeiz, Y., & Potenza, M. N. (2024). Psychometric properties of the Persian version of the cognitive emotion regulation questionnaire-short (CERQ-P-short): Reliability, validity, factor structure, treatment sensitivity, and measurement invariance. *Journal of Psychiatric Research, 170* , 1-10.	Does not contain all three domains of SR
91	Hee, P. J., Xu, Y., & Krieg, A. (2018). Validation of the Head-Toes-Knees-Shoulders task in Native Hawaiian and non-Hawaiian children. *Early Childhood Research Quarterly, 44* , 192-205.	Does not contain all three domains of SR
92	Hernández Barrios, A., & Camargo Uribe, Á. (2017). Adaptation and validation of Self-Regulation Strategy Inventory—Self-Report in university students. *Suma Psicologica, 24* (1), 9-16.	Does not contain all three domains of SR
93	Hernandez-Jorge, C. M., Rodriguez-Hernandez, A. F., Kostiv, O., Dominguez-Medina, R., Hess-Medler, S., Capote, M. C., ... & Rivero, F. (2022). Emotional Competence Assessment Scale: Teaching perspective (D-ECREA). PSICOLOGIA EDUCATIVA, 28(1), 61-69.	Not a study of psychometric properties

94	Hintermair, M., Sarimski, K., & Lang, M. (2017). Preliminary evidence assessing social–emotional competences in deaf and hard of hearing infants and toddlers using a new parent questionnaire. The Journal of Deaf Studies and Deaf Education, 22(2), 143-154.	Criteria not met
95	Hofmann, S. G., & Kashdan, T. B. (2010). The Affective Style Questionnaire: development and psychometric properties. *Journal of Psychopathology & Behavioral Assessment, 32* (2), 255-263.	Does not contain all three domains of SR
96	Howard, S. J., Neilsen-Hewett, C., de Rosnay, M., Vasseleu, E., & Melhuish, E. (2019). Evaluating the viability of a structured observational approach to assessing early self-regulation. *Early Childhood Research Quarterly, 48* , 186-197. doi:10.1016/j.ecresq.2019.03.003	Included
97	Hunter, L. J., Bierman, K. L., & Hall, C. M. (2018). Assessing noncognitive aspects of school readiness: The predictive validity of brief teacher rating scales of social–emotional competence and approaches to learning. Early Education and Development, 29(8), 1081-1094.	Criteria not met
98	Hutchison, A. N., Yeung, D. Y., Gerstein, L. H., & Wettersten, K. B. (2021). Psychometric comparison of Chinese and English versions of the Emotion Regulation Questionnaire with bilingual Hong Kong Chinese students. *International Journal of Psychology, 56* (2), 296-303.	Does not contain all three domains of SR
99	Im, G. W., Jiar, Y. K., & Talib, R. B. (2019). Development of Preschool Social Emotional Inventory for Preschoolers: A Preliminary Study. International Journal of Evaluation and Research in Education, 8(1), 158-164.	Criteria not met
100	Ioannidis, C. A., & Siegling, A. B. (2015). Criterion and incremental validity of the emotion regulation questionnaire. *Frontiers in Psychology, 6* (MAR).	Does not contain all three domains of SR
101	Ireland, M. J., Goh, H. E., & Marais, I. (2018). A rasch model analysis of the Emotion Regulation Questionnaire. Journal of Applied Measurement, 19(3), 258-270.	Mean age not <18
102	Ireland, M. J., Goh, H. E., & Marais, I. (2018). A Rasch Model Analysis of the Emotion Regulation Questionnaire. *Journal of Applied Measurement, 19* (3), 258-270.	Does not contain all three domains of SR
103	Ito, M., & Hofmann, S. G. (2014). Culture and affect: The factor structure of the affective style questionnaire and its relation with depression and anxiety among Japanese. *BMC Research Notes, 7* (1).	Does not contain all three domains of SR
104	Jamal, F., Dzulkarnain, A., Shahrudin, F., Musa, R., Sidek, S., Yusof, H., & Khalid, M. (2021). Translation, validation and cross-cultural adaptation of the Malay Emotion Regulation Checklist (ERC-M): A preliminary study. *The Medical Journal of Malaysia, 76* (5), 680-684.	Included
105	Javan, S. B., Javan, S. B., & Bashmagh, H. M. (2021). The examination of the psychometric properties of the persian version of the self-regulation questionnaire among iranian students. *Frontiers in Biomedical Technologies, 8* (1), 3-8	Does not contain all three domains of SR
106	Kahwagi, R. M., Zeidan, R. K., Haddad, C., Hallit, R., Sacre, H., Kheir, N., . . . Hallit, S. (2021). Emotion regulation among Lebanese adults: Validation of the Emotion Regulation Questionnaire and association with attachment styles. *Perspectives in Psychiatric Care, 57* (2), 809-820.	Does not contain all three domains of SR

107	Kaufman, E. A., Xia, M., Fosco, G., Yaptangco, M., Skidmore, C. R., & Crowell, S. E. (2016). The Difficulties in Emotion Regulation Scale Short Form (DERS-SF): Validation and replication in adolescent and adult samples. *Journal of Psychopathology and Behavioral Assessment, 38* , 443-455. doi:10.1007/s10862-015-9529-3	Included
108	Kerns, C. E., Comer, J. S., & Zeman, J. (2014). A preliminary psychometric evaluation of a parent-report measure of child emotional awareness and expression in a sample of anxious youth. *Cognitive Therapy and Research, 38* (3), 349-357.	Does not contain all three domains of SR
109	Kiese-Himmel, C., von Steinbuchel, N., & Gibbons, H. (2016). Assessment of Social-Communicative Behaviour Problems and Expression of Emotions in Three- to Six-Year Old Children in Nursery School and Kindergarten. *Praxis der Kinderpsychologie und Kinderpsychiatrie, 65* (3), 181-199.	Does not contain all three domains of SR
110	KKuhl, J., & Kraska, K. (1993). Self-regulation: Psychometric properties of a computer-aided instrument. German Journal of Psychology, 17, 11-24.	Articles/assessment not available in English
111	Kostiuk, L. M. (2011). Adolescent emotion regulation questionnaire: Development and validation of a measure of emotion regulation for adolescents. University of Alberta.	Dissertation
112	Kostiuk, L. M. (2013). Adolescent Emotion Regulation Questionnaire: Development and validation of a measure of emotion regulation for adolescents.DP - 2013. *Dissertation Abstracts International: Section B: The Sciences and Engineering, 74* (3-B(E))	Does not contain all three domains of SR
113	Kröner, J., Goussios, C., Schaitz, C., Streb, J., & Sosic-Vasic, Z. (2017). The construct validity of the German academic Self-Regulation Questionnaire (SRQ-A) within primary and secondary school children. *Frontiers in Psychology, 8* (JUN).	Does not contain all three domains of SR
114	Kröner, J., Goussios, C., Schaitz, C., Streb, J., & Sosic-Vasic, Z. (2017). The construct validity of the German academic Self-Regulation Questionnaire (SRQ-A) within primary and secondary school children. *Frontiers in Psychology, 8* (JUN).	Does not contain all three domains of SR
115	Lakes, K. D. (2012). The Response to Challenge Scale (RCS): The development and construct validity of an observer-rated measure of children’s self-regulation. *The International journal of educational and psychological assessment, 10* (1), 83-96.	Included
116	Lakes, K. D. (2013). Measuring self-regulation in a physically active context: Psychometric analyses of scores derived from an observer-rated measure of self-regulation. *Mental health and physical activity, 6* (3), 189-196. doi:10.1016/j.mhpa.2013.09.003	Included
117	Lan, X. (2009). Bridging naturalistic and laboratory measures of self-regulation: The development and validation of challenge tasks. University of Michigan.	Dissertation
118	Leyfer, O., John, A. E., Woodruff-Borden, J., & Mervis, C. B. (2012). Factor structure of the children's behavior questionnaire in children with williams syndrome. *Journal of Autism and Developmental Disorders, 42* (11), 2346-2353.	Does not contain all three domains of SR
119	Li, C.-H., & Wu, J.-J. (2020). Psychometric evaluation of the Chinese Version of the Emotion Regulation Questionnaire in Taiwanese college students. *Assessment, 27* (6), 1300-1309.	Does not contain all three domains of SR
120	Li, Y. M., Li, J., Zou, H., & Wei, S. (2020). Development and validation of the emotion regulation ability test for Chinese youth. *Journal of Pacific Rim Psychology, 14* .	Does not contain all three domains of SR

121	Ling, Y., He, Y., Huebner, E. S., Zeng, Y., Li, Y., & Zhao, N. (2019). Gender-Based Measurement Invariance of the Emotion Regulation Questionnaire for Children and Adolescents. *Applied Research in Quality of Life, 14* (2), 409-421.	Does not contain all three domains of SR
122	Lipska, A., Rogoza, R., Dębska, E., Ponikiewska, K., Putnam, S., & Cieciuch, J. (2022). The structure of child temperament as measured by the Polish versions of the Children's Behavior Questionnaire and the Temperament in Middle Childhood Questionnaire: insight from the network psychometrics approach. *Current Issues in Personality Psychology, 10* (4), 265-276.	Does not contain all three domains of SR
123	Liu, W., Chen, L., & Blue, P. R. (2016). Chinese adaptation and psychometric properties of the child version of the cognitive emotion regulation questionnaire. *PLoS ONE, 11* (2).	Does not contain all three domains of SR
124	Liu, W., Chen, L., & Tu, X. (2017). Chinese adaptation of Emotion Regulation Questionnaire for Children and Adolescents (ERQ-CCA): A psychometric evaluation in Chinese children. *International Journal of Psychology, 52* (5), 398-405.	Does not contain all three domains of SR
125	Liu, Y., Anderson, J. R., Weldon, A. N., Zhu, L., Sajovec, P., Pollard-Duradola, S., . . . Gonzalez, J. E. (2020). Examining the factor structure of the Child Behavior Questionnaire-Very Short Form-Teacher Form in a Spanish-speaking Mexican-American sample. *Early Childhood Research Quarterly, 53* , 403-412.	Does not contain all three domains of SR
126	Lotfi, M., Bahrampouri, L., Amini, M., Fatemitabar, R., Birashk, B., Entezari, M., & Shiasi, Y. (2019). Persian adaptation of emotion regulation questionnaire for children and adolescents (ERQ-CA). *Journal of Mazandaran University of Medical Sciences, 29* (175), 117-128.	Does not contain all three domains of SR
127	Lotfi, M., Shiasy, Y., Amini, M., Mansori, K., Hamzezade, S., Salehi, A., & Mafakhery, M. (2020). Investigating psychometric properties of interpersonal emotion regulation questionnaire (IERQ) in university students. *Journal of Mazandaran University of Medical Sciences, 30* (185), 74-85.	Does not contain all three domains of SR
128	Lubin, A. R. (2015). Validation of the Self-Regulation Strategy Inventory-Parent Rating Scale. *ProQuest LLC. Psy.D. Dissertation, Rutgers The State University of New Jersey, Graduate School of Applied and Professional Psychology* .	Does not contain all three domains of SR
129	Lucas-Molina, B., Giménez-Dasí, M., Quintanilla, L., Gorriz-Plumed, A. B., Giménez-García, C., & Sarmento-Henrique, R. (2022). Spanish validation of the Emotion Regulation Checklist (ERC) in preschool and elementary children: Relationship with emotion knowledge. *Social Development, 31* (3), 513-529. doi:10.1111/sode.12585	Included
130	Lunkenheimer, E., Kemp, C. J., Lucas-Thompson, R. G., Cole, P. M., & Albrecht, E. C. (2017). Assessing Biobehavioural Self-Regulation and Coregulation in Early Childhood: The Parent-Child Challenge Task. *Infant and Child Development, 26* (1)	Does not contain all three domains of SR
131	MacDermott, S. T., Gullone, E., Allen, J. S., King, N. J., & Tonge, B. (2010). The Emotion Regulation Index for Children and Adolescents (ERICA): a psychometric investigation. *Journal of Psychopathology & Behavioral Assessment, 32* (3), 301-314.	Does not contain all three domains of SR
132	Mantz, L. S., Bear, G. G., Yang, C., & Harris, A. (2018). The delaware social-emotional competency scale (DSECS-S): evidence of validity and reliability. Child Indicators Research, 11, 137-157.	Criteria not met

133	Martín-Albo, J., Valdivia-Salas, S., Lombas, A. S., & Jiménez, T. I. (2020). Spanish Validation of the Emotion Regulation Questionnaire for Children and Adolescents (ERQ-CA): Introducing the ERQ-SpA. *Journal of Research on Adolescence, 30* (S1), 55-60.	Does not contain all three domains of SR
134	McCoy, D. L. C., Raver, C. C., Lowenstein, A. E., & Tirado-Strayer, N. (2011). Assessing Self-Regulation in the Classroom: Validation of the BIS-11 and the BRIEF in Low-Income, Ethnic Minority School-Age Children. *Early Education and Development, 22* (6), 883-906.	Does not contain all three domains of SR
135	McCullen, J. R., Mirabile, S. P., Wuertz, S. M., & Scott, B. G. (2024). Development and adaptation of the Emotion Regulation Skills Questionnaire for adolescents. Journal of Adolescence, 96(3), 632-644.	Does not contain all three domains of SR
136	Medrano, L. A., Moretti, L., Ortiz, A., & Pereno, G. (2013). Validation of the Cognitive Emotion Regulation Questionnaire in university students of Cordoba, Argentina. *Psykhe: Revista de la Escuela de Psicologia, 22* (1), 83-96.	Does not contain all three domains of SR
137	Melka, S. E., Lancaster, S. L., Bryant, A. R., & Rodriguez, B. F. (2011). Confirmatory factor and measurement invariance analyses of the emotion regulation questionnaire. *Journal of Clinical Psychology, 67* (12), 1283-1293.	Does not contain all three domains of SR
138	Merrell, K. W., Felver-Gant, J. C., & Tom, K. M. (2011). Development and validation of a parent report measure for assessing social-emotional competencies of children and adolescents. Journal of Child and Family Studies, 20, 529-540.	Criteria not met
139	Meybodi, F. A., Mohammadkhani, P., Pourshahbaz, A., Dolatshahi, B., Mousavi, M. E., & Heydari, H. (2018). Psychometric properties of the Persian version of the Emotion Regulation Checklist. *World Family Medicine, 16* (2), 187-192. doi:10.5742/MEWFM.2018.93260	Included
140	Mohd Ali, M., Mohd Hoesni, S., Rosharudin, N. A., Yusoff, S. R., Razman, M. O. I., Khairuddin, K. F., . . . Puad Mohd Kari, D. N. (2022). Translation and Validation of the Malay Version of the Emotion Regulation Questionnaire for Children and Adolescents (ERQ-CA). *International Journal of Environmental Research and Public Health, 19* (18).	Does not contain all three domains of SR
141	Mohorić, T. (2020). Construct-related validity of the Emotional Skills and Competence Questionnaire–Children's Form (ESCQ-C). *Psychological Topics/Psihologijske teme, 29* (1), 151-166. doi:10.31820/pt.29.1.9	Included
142	Moilanen, K. L. (2007). The adolescent self-regulatory inventory: The development and validation of a questionnaire of short-term and long-term self-regulation. *Journal of Youth and Adolescence, 36* , 835-848. doi:10.1007/s10964-006-9107-9	Included
134	Molina, P., Sala, M. N., Zappulla, C., Bonfigliuoli, C., Cavioni, V., Zanetti, M. A., . . . Cicchetti, D. (2014). The Emotion Regulation Checklist - Italian translation. Validation of parent and teacher versions. *European Journal of Developmental Psychology, 11* (5), 624-634. doi:10.1080/17405629.2014.898581	Included
144	Molnár Éva, D., & Dénes, K. (2019). Hungarian version of the Children's behavior questionnaire very short form (CBQ VS). *Mentalhigiene es Pszichoszomatika, 20* (2), 159-179.	Does not contain all three domains of SR

15	Monge-López, D., Bonilla, R., & Aguilar-Freyan, W. (2017). The self-regulation strategy inventory: Spanish translation, preliminary psychometric characteristics and its relation to sociodemographic variables in sample of university students. *Avances en Psicologia Latinoamericana, 35* (1), 61-78.	Does not contain all three domains of SR
146	Monika, M., Tamas, M., Krlsztina, K.-B., & Dora, P. F. (2011). Psychometric properties of the Hungarian version of the Cognitive Emotion Regulation Questionnaire. *Psychiatria Hungarica, 26* (2), 102-111.	Does not contain all three domains of SR
147	Moreira, H., Vagos, P., Pereira, J., Fonseca, A., Canavarro, M. C., & Rijo, D. (2022). Psychometric properties of the Portuguese version of the cognitive emotion regulation questionnaire - kids version (CERQ-kids) among a sample of children and adolescents exposed to wildfires. *Current Psychology, 41* (5), 2574-2585.	Does not contain all three domains of SR
148	Moreta-Herrera, R., Dominguez-Lara, S., Rodas, J. A., Sánchez-Guevara, S., Montes-De-oca, C., Rojeab-Bravo, B., & Salinas-Palma, A. D. (2022). Examining Psychometric Properties and Measurement Invariance of the Emotion Regulation Questionnaire in an Ecuadorian Sample. *Psychological Thought, 15* (2), 57-74.	Does not contain all three domains of SR
149	Na, J. Y., Wilkinson, K., & Liang, J. (2018). Early Development of Emotional Competence (EDEC) assessment tool for children with complex communication needs: Development and evidence. American Journal of Speech-Language Pathology, 27(1), 24-36. doi:https://dx.doi.org/10.1044/2017_AJSLP-16-0058	Included
150	Naglieri, J. A., LeBuffe, P., & Shapiro, V. B. (2011). Universal screening for social–emotional competencies: A study of the reliability and validity of the DESSA-mini. Psychology in the Schools, 48(7), 660-671.	Criteria not met
151	Namatame, H., Fujisato, H., Ito, M., & Sawamiya, Y. (2020). Development and validation of a Japanese version of the emotion regulation questionnaire for children and adolescents. *Neuropsychiatric Disease and Treatment, 16* , 209-219.	Does not contain all three domains of SR
152	Navarro-Loli, J. S., Dominguez-Lara, S., & Medrano, L. A. (2020). Internal structure of the cognitive emotion regulation questionnaire (CERQ-18) in a sample of Peruvian adolescents. *Revista Iberoamericana de Diagnostico y Evaluacion Psicologica, 54* (1), 165-178.	Does not contain all three domains of SR
153	Neal, D. J., & Carey, K. B. (2005). A follow-up psychometric analysis of the self-regulation questionnaire. *Psychology of Addictive Behaviors, 19* (4), 414-422	Does not contain all three domains of SR
154	Neumann, A., van Lier, P. A., Gratz, K. L., & Koot, H. M. (2010). Multidimensional assessment of emotion regulation difficulties in adolescents using the Difficulties in Emotion Regulation Scale. Assessment, 17(1), 138-149. doi:https://dx.doi.org/10.1177/1073191109349579	Included
155	Ng, Z. J., Huebner, E. S., Maydeu-Olivares, A., & Hills, K. J. (2019). Confirmatory Factor Analytic Structure and Measurement Invariance of the Emotion Regulation Questionnaire for Children and Adolescents in a Longitudinal Sample of Adolescents. *Journal of Psychoeducational Assessment, 37* (2), 139-153.	Does not contain all three domains of SR

156	Nooripour, R., Ghanbari, N., Mozaffari, N., Ghahari, S., & Hosseini, S. R. (2023). The Persian Version of the Difficulties in Emotion Regulation Scale (DERS-18): Psychometric properties and its role in predicting aggression in iranian adolescents. *Psychological Studies, 68* (2), 236-246.	Included
157	Noronha, A. P. P., Baptista, M. N., & Batista, H. H. V. (2019). Initial psychometric studies of the emotional self-regulation scale: Adult and child-youth versions. *Estudos de Psicologia (Campinas), 36* .	Does not contain all three domains of SR
158	Nunes Baptista, M., Porto Noronha, A. P., & Bonfá-Araujo, B. (2023). Emotional Dysregulation Scale Child and Adolescent (EDEIJ): validity evidence. Ciencias Psicológicas, 17(2).	Articles/assessment not available in English
159	Ölçer, S. (2017). Validity and Reliability Study for the Social and Emotional Competence Assessment Scale among 60-72 Months-Old Children. Journal of Education and Training Studies, 5(3), 20-33.	Criteria not met
160	Olderbak, S., Uusberg, A., MacCann, C., Pollak, K. M., & Gross, J. J. (2023). The Process Model of Emotion Regulation Questionnaire: Assessing Individual Differences in Strategy Stage and Orientation. *Assessment, 30* (7), 2090-2114.	Does not contain all three domains of SR
161	Orgilés, M., Morales, A., Fernández-Martínez, I., Melero, S., & Espada, J. P. (2019). Validation of the short version of the Cognitive Emotion Regulation Questionnaire for Spanish children. *Journal of Child Health Care, 23* (1), 87-101	Does not contain all three domains of SR
162	Orgilés, M., Morales, A., Fernández-Martínez, I., Ortigosa-Quiles, J. M., & Espada, J. P. (2018). Spanish adaptation and psychometric properties of the child version of the Cognitive Emotion Regulation Questionnaire. *PLoS ONE, 13* (8).	Does not contain all three domains of SR
163	Pangestuti, R., Kadiyono, A. L., Cahyadi, S., & Agustiani, H. (2019). A modifying the instrument of self-regulation in early childhood assessment. *Jurnal Pendidikan Usia Dini, 13* (1), 114-127. doi:10.21009/10.21009/JPUD.131.09	Included
164	Park, Y. A. K. (2012). Evaluating psychometric properties of the Korean translated Social Emotional Assessment Measure for Korean preschool children. University of Oregon.	Articles/assessment not available in English
165	Pastor, M. C., López-Penadés, R., Cifre, E., & Moliner-Urdiales, D. (2019). The Spanish Version of the Emotion Regulation Questionnaire for Children and Adolescents (ERQ-CA): A Psychometric Evaluation in Early Adolescence. *Spanish Journal of Psychology* .	Does not contain all three domains of SR
166	Penner, F., Steinberg, L., & Sharp, C. (2023). The development and validation of the difficulties in emotion regulation Scale-8: Providing respondents with a uniform context that elicits thinking about situations requiring emotion regulation. *Journal of Personality Assessment, 105* (5), 657-666.	Included
167	Penza-Clyve, S., & Zeman, J. (2002). Initial Validation of the Emotion Expression Scale for Children (EESC). *Journal of Clinical Child and Adolescent Psychology, 31* (4), 540-547.	Does not contain all three domains of SR
168	Perez, J., Venta, A., Garnaat, S., & Sharp, C. (2012). The Difficulties in Emotion Regulation Scale: Factor structure and association with nonsuicidal self-injury in adolescent inpatients. *Journal of Psychopathology and Behavioral Assessment, 34* , 393-404. doi:10.1007/s10862-012-9292-7	Included

169	Perveen, A., Hidayah Abas, N. A., Hamzah, H., Kee, P., Morgul, E., Sa'ad, M., ... & Kumar, P. J. (2019). Validation of the Emotional Competency Module. Indian Journal of Public Health Research & Development, 10(7), 787-791.	Not a study of psychometric properties
170	Phillips, K., & Power, M. (2007). A new self-report measure of emotion regulation in adolescents: The Regulation of Emotions Questionnaire. *Clinical Psychology & Psychotherapy, 14* (2), 145-156. doi:10.1002/cpp.523	Included
171	Pichardo, C., Justicia, F., de la Fuente, J., Martínez-Vicente, J. M., & Berbén, A. B. (2014). Factor structure of the self-regulation questionnaire (SRQ) at Spanish Universities. The Spanish Journal of Psychology, 17, E62.	Mean age not <18
172	Pichardo, C., Justicia, F., De La Fuente, J., Martínez-Vicente, J. M., & Berbén, A. B. G. (2014). Factor structure of the Self-Regulation Questionnaire (SRQ) at Spanish universities. *Spanish Journal of Psychology, 17* (2).	Does not contain all three domains of SR
173	Pichardo, M. C., Cano, F., Garzón-Umerenkova, A., de la Fuente, J., Peralta-Sánchez, F. J., & Amate-Romera, J. (2018). Self-Regulation Questionnaire (SRQ) in Spanish adolescents: Factor structure and rasch analysis. *Frontiers in Psychology, 9* (AUG).	Does not contain all three domains of SR
174	Pinto, A., Pasian, S. R., & Malloy-Diniz, L. F. (2021). Gender invariance and psychometric properties of a Brazilian version of the Emotion Regulation Questionnaire (ERQ). *Trends in Psychiatry & Psychotherapy, 43* (2), 92-100.	Does not contain all three domains of SR
175	Potgieter, J. C. and K. F. H. Botha (2009). "Psychometric Properties of the Short Self-Regulation Questionnaire (SSRQ) in a South African Context." Journal of Psychology in Africa 19(3): 321-328.	Mean age not <18
176	Potgieter, J. C., & Botha, K. F. H. (2009). Psychometric Properties of the Short Self-Regulation Questionnaire (SSRQ) in a South African Context. *Journal of Psychology in Africa, 19* (3), 321-328	Does not contain all three domains of SR
177	Preece, D. A., Becerra, R., Robinson, K., & Gross, J. J. (2019). The Emotion Regulation Questionnaire: Psychometric Properties in General Community Samples. *Journal of Personality Assessment* .	Does not contain all three domains of SR
178	Pylypow, J., Quinn, D., Duncan, D., & Balbuena, L. (2020). A measure of emotional regulation and irritability in children and adolescents: The clinical evaluation of emotional regulation–9. *Journal of Attention Disorders* , *24* (14), 2002-2011.	Does not contain all three domains of SR
179	Quick, V., Lipsky, L., & Nansel, T. (2018). Psychometric properties and factor structure of the adapted Self-Regulation Questionnaire assessing autonomous and controlled motivation for healthful eating among youth with type 1 diabetes and their parents. *Child: Care, Health and Development, 44* (4), 651-658.	Does not contain all three domains of SR
180	Rådman, G., Claréus, B., & Daukantaitė, D. (2024). Adolescents' Emotion Regulation Strategies Questionnaire–Extended: Further development and associations with mental health problems in adolescence. *Assessment, 31* (2), 482-501.	Included
181	Ray-Yol, E., Yedidağ, E., Söğüt-Kahramanlar, M., Özdemir, A. B., & Altan-Atalay, A. (2023). Psychometric properties of interpersonal emotion regulation questionnaire in Turkish adolescents. *Current Psychology, 42* (35), 31551-31558.	Does not contain all three domains of SR

182	Reis, A. H., De Oliveira, S. E. S., Bandeira, D. R., Andrade, N. C., Abreu, N., & Sperb, T. M. (2016). Emotion Regulation Checklist (ERC): Preliminary studies of cross-cultural adaptation and validation for use in Brazil. *Temas em Psicologia, 24* (1), 97-116. doi:10.9788/TP2016.1-07	Included
183	Rice, K., Larsen, S. A., Sharp, S., & Rock, A. J. (2022). Factorial and construct validity of the Cognitive Emotion Regulation Questionnaire (CERQ) in an Australian sample. *Australian Psychologist, 57* (6), 338-351.	Does not contain all three domains of SR
184	Rice, S. M., Treeby, M. S., Gersh, E., Ogrodniczuk, J. S., & Kealy, D. (2018). The emotion regulation questionnaire: ERQ-9 factor structure and measurement invariance in Australian and Canadian community samples. *TPM - Testing, Psychometrics, Methodology in Applied Psychology, 25* (3), 369-377.	Does not contain all three domains of SR
185	Rieffe, C., Oosterveld, P., Miers, A. C., Terwogt, M. M., & Ly, V. (2008). Emotion awareness and internalising symptoms in children and adolescents: The Emotion Awareness Questionnaire revised. *Personality and Individual Differences, 45* (8), 756-761.	Does not contain all three domains of SR
186	Roberts, J. E., Tonnsen, B. L., Robinson, M., McQuillin, S. D., & Hatton, D. D. (2014). Temperament factor structure in fragile X syndrome: The Children's Behavior Questionnaire. *Research in Developmental Disabilities, 35* (2), 563-571.	Does not contain all three domains of SR
187	Rodriguez, P. R., Solar, F. C., & Navarrete, C. B. (2019). Cross-cultural adaptation of the children’s emotion regulation processes survey (CERP) in Chilean preschoolers. *Trends in Psychology, 27* , 85-97. doi:10.9788/TP2019.1-07	Included
188	Rojas-Ospina, T., & Valencia-Serrano, M. (2019). Adaptation and validation of a questionnaire on motivation self-regulation strategies in college students. *Psykhe, 28* (1), 1-15.	Does not contain all three domains of SR
189	Rosharudin, N. A., Muhammad, N. A., Mohd Daud, T. I., Hoesni, S. M., Yusoff, S. R., Razman, M. O. I., . . . Mohd Kari, D. N. P. (2023). Psychometric properties of the Malay version of the difficulties in Emotion Regulation Scale-18 in Malaysian adolescents. *Plos one, 18* (8), e0289551.	Included
190	Rothbart, M. K., Ahadi, S. A., Hersey, K. L., & Fisher, P. (2001). Investigations of temperament at three to seven years: The Children's Behavior Questionnaire. *Child Development, 72* (5), 1394-1408.	Does not contain all three domains of SR
191	Sætren, S. S., Hegelstad, W. T. V., Tjora, T., Hafstad, G. S., & Augusti, E. M. (2024). Validation of the short version of Cognitive Emotion Regulation Questionnaire for adolescents in Norway. *Scandinavian Journal of Public Health* .	Does not contain all three domains of SR
192	Sala, M. N., Molina, P., Abler, B., Kessler, H., Vanbrabant, L., & van de Schoot, R. (2012). Measurement invariance of the Emotion Regulation Questionnaire (ERQ). A cross-national validity study. *European Journal of Developmental Psychology, 9* (6), 751-757.	Does not contain all three domains of SR
193	Santos, A. C., Simões, C., Daniel, J. R., & Arriaga, P. (2023). Portuguese validation of the Cognitive Emotion Regulation Questionnaire short version in youth: Validity, reliability and invariance across gender and age. *European Journal of Developmental Psychology, 20* (2), 384-399.	Does not contain all three domains of SR
194	Sarıtaş-Atalar, D., Gençöz, T., & Özen, A. (2015). Confirmatory factor analyses of the difficulties in emotion regulation scale (DERS) in a Turkish adolescent sample. *European Journal of Psychological Assessment, 31* , 12-19. doi:10.1027/1015-5759/a000199	Included

195	Schäfer, J. L., Cibils Filho, B. R., de Moura, T. C., Tavares, V. C., Arteche, A. X., & Kristensen, C. H. (2018). Psychometric properties of the Brazilian version of the cognitive emotion regulation questionnaire. *Trends in Psychiatry and Psychotherapy, 40* (2), 160-169.	Does not contain all three domains of SR
196	Schoeps, K., Tamarit, A., Montoya-Castilla, I., & Takšić, V. (2019). Factorial structure and validity of the Emotional skills and competences Questionnaire (ESCQ) in Spanish adolescents. *Behavioral Psychology/Psicologia Conductual, 27* (2), 275-293.	Included
197	Scionti, N., Luzi, G., Zampini, L., & Marzocchi, G. M. (2023). Identifying children with self-regulation problems: Factorial structure and psychometric properties of the QUVA-p, a screening tool for preschool teachers. *European Journal of Psychological Assessment, 39* (2), 106-113.	Included
198	Šebeňa, R., Orosová, O., Petkeviciene, J., Salonna, F., Helmer, S., Lukacs, A., & Mikolajczyk, R. (2018). Psychometric evaluation of the short self-regulation questionnaire across three european countries. *Studia Psychologica, 60* (1), 5-15.	Does not contain all three domains of SR
199	Shapiro, V. B., Kim, B. K., Accomazzo, S., & Roscoe, J. N. (2016). Predictors of rater bias in the assessment of social-emotional competence. International Journal of Emotional Education, 8(2), 25-44.	Criteria not met
200	Shields, A., & Cicchetti, D. (1997). Emotion regulation among school-age children: The development and validation of a new criterion Q-sort scale. *Developmental psychology, 33* (6), 906-916. doi:10.1037/0012-1649.33.6.906	Included
201	Shum, K. K., Zheng, Q., Chak, G. S., Kei, K. T., Lam, C. W., Lam, I. K., . . . Tang, J. W. (2021). Dimensional structure of the BRIEF2 and its relations with ADHD symptoms and task performance on executive functions in Chinese children. *Child Neuropsychology, 27* (2), 165-189.	Does not contain all three domains of SR
202	Silverman, M. R., Bennett, R., Feuerstahler, L., Stadterman, J., Dick, A. S., Graziano, P., & Roy, A. K. (2022). Measuring emotion dysregulation in children with attention-deficit/hyperactivity disorder: Revisiting the factor structure of the Emotion Regulation Checklist. *Behavior therapy, 53* (2), 196-207. doi:10.1016/j.beth.2021.07.004	Included
203	Sleddens, E. F. C., Hughes, S. O., O'Connor, T. M., Beltran, A., Baranowski, J. C., Nicklas, T. A., & Baranowski, T. (2012). The Children's behavior Questionnaire very short scale: Psychometric properties and development of a one-item temperament scale. *Psychological Reports, 110* (1), 197-217.	Does not contain all three domains of SR
204	Sleddens, E. F., Kremers, S. P., Candel, M. J., De Vries, N. N., & Thijs, C. (2011). Validating the Children's Behavior Questionnaire in Dutch children: psychometric properties and a cross-cultural comparison of factor structures. *Psychological Assessment, 23* (2), 417-426.	Does not contain all three domains of SR
205	Smith-Donald, R., Raver, C. C., Hayes, T., & Richardson, B. (2007). Preliminary construct and concurrent validity of the Preschool Self-regulation Assessment (PSRA) for field-based research. *Early Childhood Research Quarterly, 22* (2), 173-187. doi:10.1016/j.ecresq.2007.01.002	Included

206	Sousa, R., Linharelhos, M., Silva, D. R. d., & Rijo, D. (2023). The difficulties in emotion regulation scale: Dimensionality, measurement invariance, and physiological correlates in community adolescents. *Journal of Clinical Psychology, 79* (10), 2351-2363.	Included
207	Spaapen, D. L., Waters, F., Brummer, L., Stopa, L., & Bucks, R. S. (2014). The emotion regulation questionnaire: Validation of the erq-9 in two community samples. *Psychological Assessment, 26* (1), 46-54.	Does not contain all three domains of SR
208	Ştefan, C. A., & Miclea, M. (2017). Reliability and Validity of Two Brief Screening Measures of Preschoolers' Social–Emotional Competencies. School Mental Health, 9, 44-65.	Criteria not met
209	Stropnik, S., & Zupancic, M. (2014). Toddler's temperament-The early childhood behavior questionnaire-Short form. *Anthropos, 46* (3-4), 85-108.	Does not contain all three domains of SR
210	Suksasilp, C., Griffiths, S., Sebastian, C. L., & Norbury, C. (2021). Reliability and validity of a temporal distancing emotion regulation task in adolescence. *Emotion, 21* (4), 830-841.	Does not contain all three domains of SR
211	Sundararajan, L., Yeh, K. H., & Ho, W. T. (2019). From Regulation to Refinement of Emotions: Indigenization of Emotion Regulation Questionnaire in Taiwan. *Journal of Theoretical and Philosophical Psychology* .	Does not contain all three domains of SR
212	Takšić, V., Mohorić, T., & Duran, M. (2009). Emotional skills and competence questionnaire (ESCQ) as a self-report measure of emotional intelligence. Horizons of Psychology, 18(3), 7-21.	Not a study of psychometric properties
213	Tanribuyurdu, T., & Yildiz, T. (2014). Preschool self-regulation assessment (PSRA): Adaptation study for Turkey. *Education and Science/Egitim ve Bilim, 39* (176), 317-328. doi:10.15390/eb.2014.3647	Included
214	Teglasi, H., Schussler, L., Gifford, K., Annotti, L. A., Sanders, C., & Liu, H. (2015). Child Behavior Questionnaire–Short Form for Teachers: Informant Correspondences and Divergences. *Assessment, 22* (6), 730-748.	Does not contain all three domains of SR
215	Teixeira, A., Silva, E., Tavares, D., & Freire, T. (2015). Portuguese validation of the Emotion Regulation Questionnaire for Children and Adolescents (ERQ-CA): relations with self-esteem and life satisfaction. *Child Indicators Research, 8* (3), 605-621.	Does not contain all three domains of SR
216	Thomas, A. E., & Muller, F. H. (2016). Development and validation of scales measuring motivational regulation for learning. *Diagnostica, 62* (2), 74-84.	Does not contain all three domains of SR
217	Thorlacius, Ö., & Gudmundsson, E. (2019). The development of the children's emotional adjustment scale–preschool version. Journal of Psychoeducational Assessment, 37(3), 263-279.	Criteria not met
218	Thorlacius, Ö., & Gudmundsson, E. (2019). The effectiveness of the children's emotional adjustment scale (ceas) in screening for mental health problems in middle childhood. School Mental Health, 11(3), 400-412.	Criteria not met
219	Tuna, E., & Bozo, Ö. (2012). The Cognitive Emotion Regulation Questionnaire: Factor Structure and Psychometric Properties of the Turkish Version. *Journal of Psychopathology & Behavioral Assessment, 34* (4), 564-570.	Does not contain all three domains of SR
220	Ulasan Ozgule, E. T., & Sumer, N. (2017). Emotion regulation during adolescence and psychological adjustment: Psychometric qualities of emotion regulation questionnaire in Turkish culture. *Turk Psikoloji Yazilari, 20* (40), 1-18.	Does not contain all three domains of SR

221	Umerenkova, A. G., Arias, J. F., Martínez-Vicente, J. M., Sevillano, L. Z., Pichardo, M. C., & García-Berbén, A. B. (2017). Validation of the Spanish Short Self-Regulation Questionnaire (SSSRQ) through Rasch analysis. *Frontiers in Psychology, 8* (MAR).	Does not contain all three domains of SR
222	Van den Bergh, B. R., & Ackx, M. (2003). A Dutch version of Rothbart's Children's Behavior Questionnaire. Internal consistency and threefactor-model of the scales. *Kind en Adolescent, 24* (2), 77-84.	Does not contain all three domains of SR
223	Victor, S. E., & Klonsky, E. D. (2016). Validation of a brief version of the Difficulties in Emotion Regulation Scale (DERS-18) in five samples. *Journal of Psychopathology and Behavioral Assessment, 38* , 582-589. doi:10.1007/s10862-016-9547-9	Included
224	Villacura-Herrera, C., Gaete, J., Andaur, J., Meza, D., Robinson, J., & Núñez, D. (2023). Evidence for validity, reliability and measurement invariance of the emotion regulation questionnaire for children and adolescents (ERQ-CA) in secondary students from Chile. *Current Psychology, 42* (31), 27771-27782.	Does not contain all three domains of SR
225	Wang, D., Yuan, B., Han, H., & Wang, C. (2022). Validity and reliability of emotion regulation questionnaire (ERQ) in Chinese rural-to-urban migrant adolescents and young adults. *Current Psychology, 41* (4), 2346-2353.	Does not contain all three domains of SR
226	Wang, J., Luo, X., Liu, Q., Peng, W., Liu, Z., Ge, Z., . . . Zhong, M. (2023). Factorial invariance of the cognitive emotion regulation questionnaire across gender in Chinese college students. *Current Psychology, 42* (12), 9879-9889.	Does not contain all three domains of SR
227	Wang, J., Xu, W., Fu, Z., Yu, W., He, L., Sun, L., . . . Hofmann, S. G. (2019). Psychometric properties of the Chinese version of the Affective Style Questionnaire and its role as a moderator of the relationship between stress and negative affect. *Journal of Health Psychology, 24* (5), 613-622.	Does not contain all three domains of SR
228	Wang, L. F., Wei, M., Chang, J. H., & Chiao, H. (2023). The Children's Dual Emotion Regulation Strategy scale: An integrated perspective of Western and East Asian cultures. Asian Journal of Social Psychology, 26(4), 461-474.	Does not contain all three domains of SR
229	Wang, S., Liu, C., Byrne, E. M., & Xie, H. (2024). Measuring preschoolers' behavioral self-regulation in the contexts of child–adult interactions. *Current Psychology* , *43* (16), 14523-14537.	Unable to retrieve assessment items
230	Wei, Y.-M., & Liu, Y.-X. (2008). Reliability and validity of the Cognitive Emotion Regulation Questionnaire (CERQ) in Chinese college students. *Chinese Mental Health Journal, 22* (4), 281-284	Does not contain all three domains of SR
231	Weinberg, A., & Klonsky, E. D. (2009). Measurement of emotion dysregulation in adolescents. *Psychological Assessment, 21* (4), 616-621. doi:10.1037/a0016669	Included
232	Xie, D., Lu, J., & Xie, Z. (2015). Online emotion regulation questionnaire for adolescents: Development and preliminary validation. Social Behavior and Personality: an international journal, 43(6), 955-965.	Articles/assessment not available in English
233	Yeo, G. B. and E. R. Frederiks (2011). "Cognitive and Affective Regulation: Scale Validation and Nomological Network Analysis." Applied Psychology: An International Review 60(4): 546-575.	Mean age not <18
234	Zhang, Y., & Bian, Y. (2020). Emotion Regulation Questionnaire for Cross-Gender Measurement Invariance in Chinese University Students. *Frontiers in Psychology, 11* .	Does not contain all three domains of SR

235	Zhao, M., Kuan, G., Zhou, K., Musa, R. M., Abdul Majeed, A. P. P., & Kueh, Y. C. (2024). Psychometric properties and gender invariance of the 8-item emotion regulation questionnaire (ERQ-8) among Chinese university students. *PLoS ONE, 19*	Does not contain all three domains of SR
236	Zhao, X., Zhang, B.-r., Zhang, P., Pan, L., & Zhou, R.-l. (2015). Reliability and validity of Emotion Regulation Questionnaire in middle school students. *Chinese Journal of Clinical Psychology, 23* (1), 22-25.	Does not contain all three domains of SR
237	Zhao, Y., Li, P., Wang, X., Kong, L., Wu, Y., & Liu, X. (2020). The Chinese version of the behavioral emotion regulation questionnaire: Psychometric properties among university students. *Neuropsychiatric Disease and Treatment, 16* , 1889-1897.	Does not contain all three domains of SR
238	Zhou, Y., Bullock, A., Liu, J., Fu, R., Coplan, R. J., & Cheah, C. S. (2016). Validation of the self-regulation scale in Chinese children. Journal of Psychoeducational Assessment, 34(6), 589-594.	Criteria not met
239	Zhou, Y., Daukantaitė, D., Lundh, L.-G., Wångby-Lundh, M., & Ryde, A. (2020). Adolescents' emotion regulation strategies questionnaire: Initial validation and prospective associations with nonsuicidal self-injury and other mental health problems in adolescence and young adulthood in a Swedish youth cohort. *Frontiers in psychiatry, 11* , 462. doi:10.3389/fpsyt.2020.00462	Included
240	Zhou, Y., Ji, X.-q., & Liu, J.-s. (2014). Reliability and validity of the Chinese version of the Academic Self-regulation Questionnaire. *Chinese Journal of Clinical Psychology, 22* (2), 256-259	Does not contain all three domains of SR
241	Zhu, X.-Z., Luo, F.-S., Yao, S.-Q., Auerbach, R. P., & Abela, J. R. (2007). Reliability and validity of the Cognitive Emotion Regulation Questionnaire-Chinese version. *Chinese Journal of Clinical Psychology, 15* (2), 121-124	Does not contain all three domains of SR
242	Zhu, X., Auerbach, R. P., Yao, S., Abela, J. J. R. Z., Xiao, J., & Tong, X. (2008). Psychometric properties of the Cognitive Emotion Regulation Questionnaire: Chinese version. *Cognition and Emotion, 22* (2), 288-307	Does not contain all three domains of SR
