# Supplementary material for: A systematic review of self-regulation measures in children: Exploring characteristics and psychometric properties
Source: PLoS One. 2024 Sep 19;19(9):e0309895. doi: 10.1371/journal.pone.0309895 (PMC11412528; doi:10.1371/journal.pone.0309895)
Supplement: S4 File — (PDF) [file pone.0309895.s004.pdf]

Quality assessment of Included Studies

Study	Kmet Checklist Item													
	1	2	3	4	5	6	7	8	9	10	11	12	13	14
Aslan and Sevincler-Togan [31]	2	2	2	1	X	X	X	1	2	2	2	1	2	1
Bartroli, Angulo-Brunet [51]	2	2	1	2	X	X	X	2	2	2	2	2	2	2
Bassett, Denham [70]	2	2	2	2	X	X	X	1	2	2	2	1	2	2
Bowie [37]	2	2	2	1	X	X	X	1	2	2	1	2	2	1
Bunford, Dawson [50]	2	1	1	1	X	X	X	2	2	2	2	2	2	2
Burney and Kromrey [30]	2	2	1	1	X	X	X	2	2	2	1	1	1	2
Charak, Byllesby [44]	2	2	2	2	X	X	X	1	2	2	2	1	2	1
Chennaz, Valente [60]	2	2	2	2	X	X	X	1	2	2	1	1	2	2
Costa, Faria [61]	2	2	2	2	X	X	X	2	2	2	2	1	2	1
Daneri, Sulik [69]	2	2	2	2	X	X	X	2	2	2	2	1	2	2
Danisman, Dereli [57]	2	2`	1	2	X	X	X	1	2	2	2	1	2	2
Demirpence Secinti and Sen [45]	2	2	1	2	X	X	X	1	2	2	1	1	2	2
Dias, del Castillo [35]	2	2	2	1	X	X	X	2	2	2	2	1	2	1
Faria and Lima-Santos [64]	2	2	2	1	X	X	X	2	2	2	2	2	2	1
Faria, Lima Santos [62]	2	2	1	1	X	X	X	2	2	2	2	1	2	2
Gajda, Małkowska-Szkutnik [76]	2	2	1	2	X	X	X	1	1	2	2	1	2	2
Howard, Neilsen-Hewett [71]	2	2	1	1	X	X	X	1	2	2	1	1	2	2
Jamal, Dzulkarnain [29]	1	1	1	1	X	X	X	2	1	1	2	1	1	2
Kaufman, Xia [49]	2	2	2	2	X	X	X	1	1	2	2	2	2	2
Lakes [73]	2	2	2	1	X	X	X	2	2	2	1	1	2	2
Lakes [74]	2	2	1	1	X	X	X	2	2	2	2	1	2	1
Lucas-Molina, Giménez-Dasí [58]	2	2	2	2	X	X	X	2	2	1	2	2	2	2
Meybodi, Mohammadkhani [56]	1	1	1	1	X	X	X	2	2	1	2	2	1	2
Mohorić [65]	2	2	1	2	X	X	X	1	2	2	2	1	2	2
Moilanen [34]	2	2	2	2	X	X	X	1	2	1	2	2	2	1
Molina, Sala [54]	1	2	1	1	X	X	X	2	2	1	2	2	1	2
Na, Wilkinson [52]	2	2	1	1	X	X	X	1	2	2	1	1	1	2
Neumann, van Lier [39]	2	2	2	2	X	X	X	2	2	1	2	1	2	2
Nooripour, Ghanbari [47]	2	2	2	1	X	X	X	1	2	2	2	1	2	2
Pangestuti, Kadiyono [68]	1	2	1	1	X	X	X	2	2	2	2	1	2	1
Penner, Steinberg [43]	2	2	2	2	X	X	X	2	2	2	1	1	2	2
Perez, Venta [41]	2	2	2	2	X	X	X	2	2	2	2	1	2	2
Phillips and Power [75]	2	2	1	1	X	X	X	2	1	2	2	1	2	1
Rådman, Claréus [33]	2	2	2	1	X	X	X	2	2	2	2	1	2	2
Reis, De Oliveira [55]	2	2	2	2	X	X	X	2	2	1	2	1	2	2
Rodriguez, Solar [36]	2	2	2	2	X	X	X	2	2	2	2	1	2	2
Rosharudin, Muhammad [46]	2	2	2	2	X	X	X	1	2	2	2	1	2	2
Sarıtaş-Atalar, Gençöz [40]	2	2	2	2	X	X	X	1	2	2	2	1	2	1
Schoeps, Tamarit [63]	2	2	2	2	X	X	X	2	2	2	2	1	2	1
Scionti, Luzi [72]	2	2	1	1	X	X	X	1	2	2	2	2	2	1

Study	Kmet Checklist Item													
	**1**	**2**	**3**	**4**	**5**	**6**	**7**	**8**	**9**	**10**	**11**	**12**	**13**	**14**
Shields and Cicchetti [53]	1	2	1	2	X	X	X	2	2	2	2	1	2	2
Silverman, Bennett [59]	2	2	2	2	X	X	X	2	2	2	2	1	2	2
Smith-Donald, Raver [66]	2	2	1	1	X	X	X	2	2	2	2	1	2	2
Sousa, Linharelhos [42]	2	2	2	2	X	X	X	1	2	2	2	1	2	2
Tanribuyurdu and Yildiz [67]	2	2	2	1	X	X	X	2	2	2	2	1	2	2
Victor and Klonsky [48]	2	2	2	2	X	X	X	1	2	2	1	1	1	1
Weinberg and Klonsky [38]	2	2	1	1	X	X	X	2	2	2	1	1	2	2
Zhou, Daukantaitė [32]	2	2	2	1	X	X	X	2	2	2	2	1	2	2
